# PET Radiopharmaceuticals for Alzheimer’s Disease and Parkinson’s Disease Diagnosis, the Current and Future Landscape

**DOI:** 10.3390/molecules25040977

**Published:** 2020-02-21

**Authors:** Bright Chukwunwike Uzuegbunam, Damiano Librizzi, Behrooz Hooshyar Yousefi

**Affiliations:** 1 Nuclear Medicine Department, and Neuroimaging Center, Technical University of Munich, 81675 Munich, Germany; b.uzuegbunam@tum.de; 2 Department of Nuclear Medicine, Philipps-University of Marburg, 35043 Marburg, Germany; librizzi@med.uni-marburg.de

**Keywords:** Alzheimer’s disease, Parkinson’s disease, β-amyloid plaques, neurofibrillary tangles, α-synucleinopathy, positron emission tomography (PET), diagnostic imaging probes

## Abstract

Ironically, population aging which is considered a public health success has been accompanied by a myriad of new health challenges, which include neurodegenerative disorders (NDDs), the incidence of which increases proportionally to age. Among them, Alzheimer’s disease (AD) and Parkinson’s disease (PD) are the most common, with the misfolding and the aggregation of proteins being common and causal in the pathogenesis of both diseases. AD is characterized by the presence of hyperphosphorylated τ protein (tau), which is the main component of neurofibrillary tangles (NFTs), and senile plaques the main component of which is β-amyloid peptide aggregates (Aβ). The neuropathological hallmark of PD is α-synuclein aggregates (α-syn), which are present as insoluble fibrils, the primary structural component of Lewy body (LB) and neurites (LN). An increasing number of non-invasive PET examinations have been used for AD, to monitor the pathological progress (hallmarks) of disease. Notwithstanding, still the need for the development of novel detection tools for other proteinopathies still remains. This review, although not exhaustively, looks at the timeline of the development of existing tracers used in the imaging of Aβ and important moments that led to the development of these tracers.

## 1. Introduction

Of all the causes of dementia, AD stands in first place and makes up the largest part—about two-thirds—of all differential diagnoses [1,2,3], and it is the most common form of dementia in persons older than 65 years [4]. Others have vascular dementia, mixed dementia, PD, Lewy body dementia (LBD) or frontotemporal degeneration (FTD) [2]. Although AD and PD present markedly different clinical and pathological features, many mechanisms involved in AD and PD may be the same, such as mutation in genes, the roles of α-synuclein and tau protein aggregates in oxidative stress and mitochondrial dysfunction, dysregulation in the brain homeostasis of iron [5].

The WHO in 2012 named the prevention and control of neurocognitive disorders (mild cognitive impairment (MCI) or Alzheimer’s type dementia) a global public health priority. As of 2012, it was estimated that worldwide 35.6 million people are living with dementia. By 2030 this number will double and by 2050 triple [3]. The World Alzheimer Report also in 2018 estimated that there are 50 million people in the world with dementia. This number by 2050 is likely to rise to about 152 million people [2] a projection not far from that made by the WHO way back in 2012.

In the pathogenesis of AD two proteins are implicated β-amyloid peptide aggregates (Aβ) and tau. Based on several scientific evidences, AD is histopathologically characterized by the progressive deposition of Aβ peptides into the interneuronal space [2,6,7]. The pathogenic pathways leading to AD involve several mechanisms which include the dysfunction of cholinergic neurons and the aggregation of tau, however, it has been shown that the amyloid cascade plays a significant role.

The amyloid cascade assumes that the pathogenesis of AD is as a result of a dysfunction in the synthesis and the secretion of the amyloid precursor protein (APP), usually cleaved by the proteases in the secretase family. Normally, the cleavage of APP by α-secretase within the Aβ domain releases soluble APP-α which is non-pathologic, whereas, in pathology, Aβ is generated from APP via successional cleavages by β-secretase followed by the γ-secretase complex, which cuts the γ-site of the carboxyl-terminal fragment of APP producing two major Aβ isoforms: Aβ_1-42_ and Aβ_1-40_, which subsequently aggregate to form β-amyloid plaques [8,9]. Aβ_1-42_ comprises a major part of amyloid plaques owing to its low solubility and tendency to form aggregates with β-pleated sheet structure [9].

Neurodegeneration and neuronal dysfunction are caused by the binding of extracellular Aβ oligomers to the neuronal surface, leading to functional disruption of a number of receptors, finally culminating in dysfunction and neurodegeneration [2,10]. The accumulation of hyperphosphorylated tau protein in neurons, which normally is a microtubule-associated protein (MAP) abundantly expressed in the central nervous system, is another key player in the pathogenesis of AD. As a result of abnormal hyperphosphorylation the protein self-aggregates and forms paired helical filaments (PHF), which leads to the formation of intracellular neurofibrillary tangles, which ultimately block the neuronal transport system [2,11,12].

A definitive diagnosis of AD still requires a histological examination of post-mortem brain sample [13,14,15]. However, in living patient’s cerebrospinal fluid (CSF) biomarkers and positron emission tomography (PET), in combination with several new clinical criteria can assist in the diagnosis [16,17], and for symptomatic patients with familial early-onset AD, it is recommended to undergo clinical genetic testing together with their asymptomatic relatives [18,19,20].

The European Medicines Agency has presented the measurement of Aβ peptides and total tau protein levels in the CSF as a complementary usable tool in the diagnosis and monitoring of AD [21,22]. Albeit a less expensive method of evaluation, the method is invasive and carries the risks of adverse effects and discomfiture associated with a lumbar puncture [23,24,25].

Non-invasive modern imaging techniques allow to identify either patients who are at risk of developing AD, and also to monitor disease progression or both [26,27,28]. Positron emission tomography (PET) imaging especially, which is superior to other imaging techniques in terms of sensitivity, since only picomolar concentrations of the radiotracers are required allows to visualize, characterize and quantify physiological activities at molecular and cellular levels [29,30]. Hence, it may serve as an important diagnostic tool in the field of drug discovery and development, in order to monitor disease progression and the interaction of ligands with their targets.

Aβ is the most studied and first target for the neuroimaging of AD [31], hence it is no surprise that there are already selective PET radiotracers for its imaging. In 2003, Mathis et al. reported the carbon-11 labeled Pittsburgh compound B ([^11^C]PiB), and the first successful Aβ-selective PET radioligand, which is a derivative of thioflavin (Th-T) an amyloid-binding histological fluorescent dye [32,33].

The discovery of [^11^C]PiB led to further tracer development of other Aβ tracers. Three of which are already FDA approved and are ^18^F-labeled [27] (a radioisotope with a relatively longer half-life of 109.7 min [34], in comparison to carbon-11 with a shorter half-life of 20.3 min, a property that logistically limits its use to centers with cyclotron on-site [35]): [^18^F]florbetaben (Neuraceq) [36]; [^18^F]florbetapir (Amyvid) [37]; [^18^F]flutemetamol (Vizamyl) [38].

So far, there are other findings that the density and neocortical spread of NFTs correlate better with neurodegeneration and cognitive decline in AD patients [39,40,41,42], in spite of Aβ pathology temporarily preceding tau pathology [27]. Recent evidence further corroborates initial findings of the dominant role of tau in the pathogenesis of AD [39,43,44], backing this protein as a diagnostic as well as a therapeutic target [45].

Moreover, since apart from AD there are other NDD associated with amyloid pathology, amyloid imaging is not enough to differentiate dementia subtypes [27]. Nevertheless, NFTs are also present in other dementias, like FTD, some neurodegenerative movement disorders like corticobasal degeneration (CBD) and progressive supranuclear palsy (PSP) [45]. More recently, Vanhaute et al. reported that the loss of synaptic density in the medial temporal lobe is linked to an increased tau deposition in AD [46,47]. Hence, a radiotracer, that could quantify NFTs would help to understand the pathophysiology and clinical management not only of AD, but these other NDD. Furthermore, when done in conjunction with amyloid diagnosis, PET imaging of NFTs might provide a way to distinguish between AD dementia (when there are NFTs and Aβ present) and non-AD dementia (when NFTs and Aβ are absent). Furthermore, the application of Aβ imaging is just approved for the exclusion of AD in patients with cognitive impairment but amyloid PET-negative [48]. Also, it is being evaluated as a diagnostic tool for the definition of the preclinical stages of AD [49]. Due to the abovementioned reasons, several academic and industrial groups are currently making efforts to develop tau aggregate tracers, which are not only selective, but also with minimal or no off-target binding [50,51,52,53].

The α-synucleinopathies: PD, LBD, multiple system atrophy (MSA) have their pathological hallmark as α-syn aggregates included in Lewy body (LB), Lewy neurites (LN), and glial cytoplasmic inclusions (GCI) in MSA [54,55,56,57]. α-Synuclein is a small (140 amino acid residues) highly soluble presynaptic protein that normally exists in a native unfolded state. In PD, there is formation of highly ordered insoluble aggregates known as α-syn fibrils, which are stabilized by β-sheet protein structure [58,59,60,61].

The identification of point mutations in the SNCA gene in familial cases of PD nearly 23 years ago first linked α-syn to PD [62], and this was corroborated by the additional discovery that increased genetic copies of α-synuclein in the form of duplications and triplications of the SNCA gene are enough to cause PD; the higher gene copy, the earlier the age of disease onset and the more severe the disease [63,64,65]. More recently, further investigation into the genetic aspects of the disease culminated in genome-wide association studies (GWAS), and candidate gene association studies which have repeatedly validated that statistically relevant signals linked to PD are common variants near the SNCA, LRRK2, MAPT and low-frequency coding variants in GBA (glucocerebrosidase) genes [66]. Moreover, in GWAS so far, not less than 41 risk loci for PD have been identified [67,68]. Even in the sporadic forms of the disease, α-syn as a candidate risk gene has shown significant associations between variation within the SNCA gene and a higher risk of developing PD [69].

It has been known for some time now based on fairly strong evidence that the motor phase of classical PD occurs after a premotor period that could last for a considerable number of years if not decades [70]. Before the appearance of motor symptoms, at least 50% of substantia nigra (stage 3 of the Braak staging) cells have to be lost [71,72] and likely a loss of a higher percentage of dopaminergic nerve endings in the putamen [73]. Based on the findings of Braak et al., there are 6 stages in which the deposition of α-syn in LBs and LNs occurs sequentially and additively [74]. Overall, it is evident that pathophysiological changes in the central nervous system in PD involves the abnormal deposition of α-syn occurs early in PD, hence the earliest definition and most precise detection of premotor PD should be based on the imaging of aggregate α-syn, not dopaminergic alterations.

Despite the high abundance of α-syn in the nervous system, where it constitutes 1% of all cytosolic proteins [75], the amount of α-syn aggregates, however, in LBD and MSA brain is 10-fold or lower than that of Aβ in AD brain, and in advanced cases in the range of 50–200 nM in brainstem and subcortical regions, and moreover, they typically have a small size, which complicates detections [76,77].

Unlike Aβ, but similar to NFTs, LBs are intraneuronal and GCI are intraglial, hence any tracer for the detection α-syn must readily pass through the blood-brain barrier (BBB), and subsequently the cell membrane to access its target [77,78]. Unfortunately, due to the structural similarity of β-pleated sheets amongst different species of amyloid fibrils, and the colocalization of α-syn aggregates with other aggregating amyloid proteins like Aβ plaque and tau fibrils tracer, selectivity for α-syn aggregates over the others is a desired quality. This explains why non-selective ligands are more common than selective tracers [79,80,81,82].

Generally, good PET radiotracers for brain amyloid imaging should have the qualities prerequisite for successful central nervous system ligands [83,84]. A good brain penetration via passive diffusion, relatively small molecular weight (< 700 Da), moderate lipophilicity 1–3 at physiological pH (7.4), lack of P-glycoprotein substrate activity, lack of BBB permeable radioactive metabolites or intracerebral radiometabolites, etc. Most importantly, they should with high affinity selectively and reversibly bind to targets in the brain. Target selectivity an important trait depends on factors such as the relative affinities of the tracer to target (specific binding) and non-target (non-specific binding) sites, its brain distribution and the relative concentration of the binding sites. Both target and non-target binding sites should be considered when developing a brain tracer [77,81,82,85,86].

Additionally, a slow and reversible off-rates coupled (k_off_) with relatively high on-rates (k_on_), which is reflected by an equilibrium dissociation constant (K_d_) in the range of 1 nM. A low K_d_ value in the nanomolar (nM) range could guarantee that the radioligand-amyloid complex remains intact long enough for a washout of non-specifically bound tracers to occur, hence allowing good signal-to-noise contrast. It is also needed especially when dealing with short-lived PET radioisotopes like ^11^C with a half-life of 20.3 min and ^18^F half-life 109.8 min. A standard uptake value (SUV) in the brain > 1.0 within a few min of intravenous injection is also required. Large molecules, antibodies, and nanobodies can cross the BBB, however they are unable to attain an SUV value > 1.0 a few min post-injection (p.i), and this has been a disqualifying criterion for large ligands labeled with short-lived radioisotopes [81,85,86].

## 2. PET Imaging Agents for the Diagnosis AD and PD

### 2.1. PET-Tracers for the Imaging of Aβ Plaques

#### 2.1.1. First Generation of Aβ PET Tracers

##### Benzothiazole (BTA) Derivatives

**Figure 1:**
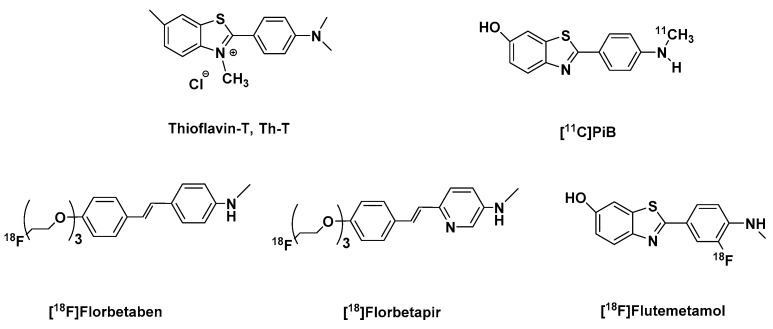
Structures of thioflavin-T, [^11^C]PiB, and the FDA approved Aβ-PET tracers: [^18^F]florbetaben, [^18^F]florbetapir, and [^18^F]flutemetamol.

The development of amyloid-specific imaging compounds is based mostly on conjugated dyes like Th-T (Figure 1) and Congo red, that are used in postmortem AD brain sections for the staining of plaques and tangles [87,88,89,90]. The synthesis of the hundreds of the derivatives of the latter by the Pittsburgh group gave rise to a series of pan-amyloid imaging agents that showed nanomolar binding affinities for Aβ, tau, α-syn, and prion aggregates. Notwithstanding, a number of these compounds ionize at physiological pH, and for this reason did not achieve high brain uptake ( > 1 SUV) a few min post intravenous injection [32,91].

**Figure 2:**
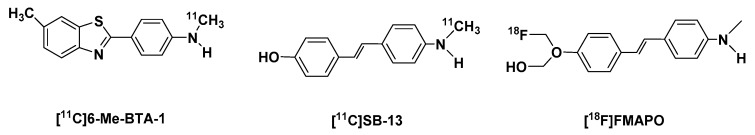
Structures of the predecessors of FDA approved Aβ-PET tracers: [^11^C]6-Me-BTA-1, [^11^C]SB-13, [^18^F]FMAPO.

The examination of the derivatives of Th-T derivatives followed: making the dye neutral by the removal of the methyl group attached to the benzothiazole ring via the nitrogen atom of the ring, hence the positive charge on the benzothiazole ring gave rise to compounds (known as benzothiazole anilines or BTAs) with improved lipophilicity, [^11^C]6-Me-BTA-1 (Figure 2) being the best in the series. It was 6-fold more lipophilic, and readily crossed the BBB in brains of rodents, and showed 44-fold more affinity for synthetic Aβ fibrils (Table 1) than did Th-T [92,93].

Further manipulation of the benzothiazole ring by derivatizing the C-6 position and varying the degree of methylation of the aniline nitrogen gave a series of ligands with high affinity for Aβ fibrils. Of these radiotracers, the monomethylated-aniline derivative ([^11^C]PiB [^11^C]6-OH-BTA-1 (Figure 1), was selected (which will be referred to as just PiB throughout the paper). It showed a combination of favorable pharmacokinetics as PiB, the highest brain clearance 5 times faster than at 30 min and a high binding affinity to Aβ plaques approximately 207-fold than Th-T [94] (Table 1), with a very low binding affinity to aggregated tau, with a ratio of tau-to-Aβ ((K_itau_/K_iAβ_) greater than 100-fold [33,95,96,97].

Clinical study with PiB showed that AD patients retained PiB in areas of association cortex known to contain large amounts of amyloid deposits [33]. Further clinical studies to confirm if there is abnormal binding of PiB in clinically healthy individuals showed that PiB-PET not only was able to detect Aβ deposits in AD patients but also in some nondemented patients, hence suggesting that amyloid imaging might be useful in the detection AD in its preclinical stages [98]. Additionally, it was confirmed that there is a direct correlation of the retention of PiB in vivo with region-matched quantitative analyses of Aβ plaques in the same patient, upon post-mortem examination of clinically diagnosed and autopsy-confirmed AD subjects [99]. This too additionally validated PiB-PET as a method for evaluating the amyloid plaque burden in AD subjects [33].

In an experiment carried out by Serdons et al. it was discovered that more than 80% of the tracer remains intact 60 min p.i [100,101]. The radiometabolites of PiB found in animal and human blood, due to their high polarity did not easily pass through the BBB [94,100]. One of the identified radiometabolites 6-sulfato-O-PiB, and others produced in rat brain, built up over time and complicated pharmacokinetic analyses [95,102]. Fortunately, the intracerebral metabolism of PiB is limited only to rats and was not observed in mice, humans, and other nonhuman primates [95].

The success of PiB for in vivo imaging of Aβ plaque deposition led to the development of an ^18^F analog, which would perform similarly. The development of ^18^F-labeled radiotracers for the imaging of amyloid deposits in AD was on the basis that, as previously mentioned, carbon-11 with which PiB was labeled has a half-life 20.3 min, and this limits its use to PET centers with cyclotron on-site and with experience in ^11^C-radiochemistry [33,36].

A variety of structural analogs were developed and evaluated both in vitro [103] and preclinically, out of which flutemetamol also known as [^18^F]GE067 ([^18^F]3′F-PiB) (Figure 1) was selected [104]. In vivo studies in rats and mice showed that it has similar pharmacokinetics as PiB. They both readily entered the brain, however, flutemetamol which is more lipophilic was washed out more slowly from the brain approximately 1.4 times slower (Table 1), especially from the white matter [105].

Initial human studies, in which flutemetamol and PiB were compared in AD and control subjects, the former showed similar uptake and specific binding attributes as PiB [104]. A phase-III trial demonstrated that it is safe with high specificity and sensitivity for the in vivo detection of brain Aβ density [106,107]. It was approved by the FDA in 2013 [108].

##### The Stilbene and Styrylpyridine Derivatives

The discovery of [^3^H]SB-13, a stilbene derivative which showed a high binding affinity to postmortem AD brain homogenates [109], led to subsequent labeling with carbon-11 to afford [^11^C]SB-13 (4 methylamino-4′-hydroxystilbene) (Figure 2). The tracer displayed a good brain uptake and brain clearance (Table 1) [110]. In vivo human PET-imaging it displayed properties similar to PiB in discriminating between AD and non-AD patients [111].

The similarities between PiB and SB-13 in addition to their similar biological properties are also in their chemical structures: the presence of a highly conjugated aromatic ring with an electron-donating group (N-methylamine (-NHCH_3_) or hydroxyl (-OH)) at the end of the molecule and the relative planarity of both ligands [90].

Early attempts at the development of ^18^F-labeled SB-13 was unsuccessful, due to the high lipophilicity and high nonspecific binding in the brain shown by [^18^F]SB-13 derivatives with a fluoroalkyl group on either ends of their structures. In order to reduce the lipophilicity of the ligands, the stilbene scaffold was further modified by the introduction of different functional groups. Based on in vitro and in vivo biological assays a NH-CH_3_ derivative [^18^F]FMAPO, with a 2-fluoromethyl-1,3-propylenediol group tethered to the phenol end of molecule (Figure 2) was selected for not only exhibiting a selectivity and specific binding to Aβ plaques in AD brain homogenate binding studies but also for showing a higher brain penetration in 2 min, which was nearly three times higher than that of flutemetamol in 5 min (Table 1). Although it displayed a slower washout than the latter, at 60 min p.i. the concentration in the brain was less than 1%ID/g [103,112].

In order to circumvent the complication of in vivo metabolism, which might result due to the presence of a chiral center in the fluorine containing side chain, another series of stilbene derivatives were synthesized with polyethylene glycol (PEG) units of different lengths (n = 2–12) tethered to the 4′-OH group, with ^18^F attached at the end of PEG side-chain. This also provided a way to maintain a small molecular weight, adjust lipophilicity and facilitate a simple ^18^F-labeling by nucleophilic substitution. Structure-activity relationship (SAR) studies showed that high binding affinity was maintained when n < 8, and from 8 and above there was a significant reduction in binding affinity. There was a noticeable decrease in brain penetration as shown by in vivo biodistribution studies when n > 5 [113,114,115], perhaps partly due to increased molecular weight and total polar surface area (tPSA).

Of the four ligands which performed well in in vitro and in vivo assays, florbetaben (Figure 1) also known as AV-1, or BAY94-9172 with n = 3, was selected. Although the tracer did not have the highest affinity for Aβ in comparison with its structural analogs or the fastest washout rate (Table 1) from the brain of healthy mice [114], it, however, showed selectivity for Aβ and non-appreciable binding to NFTs, Pick bodies, LBs and GCIs [112]. Furthermore, binding to postmortem cortex of subjects with FTD or postmortem brain tissue from other NDDs like tauopathies and α-synucleinopathies was not observed [114]. With no observable effects at 100x the expected human dose in preclinical toxicity studies in a different animal species, florbetaben was deemed suitable for human studies [116]. In 2014, it was approved by the FDA [117].

In order to obtain an Aβ tracer with improved in vivo biological properties of targeting Aβ plaques, so that a high signal to noise ratio is quickly and more efficiently achieved, some critical and competing factors were taken into consideration: initial brain uptake, washout from non-afflicted brain regions, in vivo metabolism, and optimal time in the accomplishment of the highest target-to-non-target ratio. For this reason, the stilbene ring was further explored. The fluoropegylation discussed above was extended from stilbene to styrylpyridine series. This was achieved by exchanging one of the stilbene benzene rings for a pyridine ring. This led to the development of florbetapir also known as [^18^F]AV45 [118]. It displayed 2-fold more binding affinity to Aβ in postmortem AD brain homogenates than florbetaben. Nevertheless, it showed a slightly lower initial uptake and washout rate from the brain of healthy mice than florbetaben (Table 1) [114,119].

An initial clinical trial with a tertiary amine derivative, which was similar to florbetapir but for the dimethylation of the aniline nitrogen suggested lower than expected brain uptake, probably due to a fast in vivo metabolism by N-demethylation. Of all the evaluated ligands, faster brain kinetics was exhibited more by florbetapir, and it also displayed an excellent brain uptake and washout in humans. The signal to noise ratio in the brain approaches an optimal level in 40–60 min post intravenous injection. In vitro metabolic stability assay also demonstrated that it is more stable towards microsomal degradation than florbetaben [115,118].

In AD patients, florbetapir from 30 min p.i. showed a clear separation between cortical and cerebellar activity, hence making it possible to start brain PET scan 30–50 min p.i. [120]. Significant elevations of tracer uptake in several brain regions of AD patients in comparison with controls were observed upon visual evaluation and analysis using semiquantitative methods. Results from phase III clinical trial showed a distinct correlation between the distribution of Aβ and florbetapir PET images at postmortem examination. Furthermore, no serious side effects were recorded in any of the clinical trials of the tracer [121]. It was approved by FDA in 2012 [31,122].

#### 2.1.2. Second Generation of Aβ PET Tracers

##### Benzofuran, Benzoxazole and Imidazobenzothiazole Derivatives

**Figure 3:**
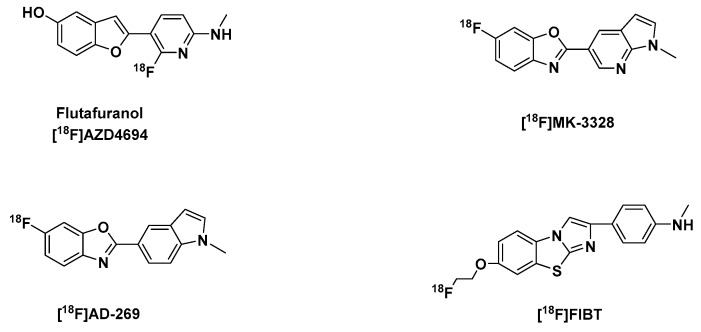
Structures of second generation Aβ-PET tracers: [^18^F]AZD4694, [^18^F]MK-3328, [^18^F]AD-269, [^18^F]FIBT.

Other notable Aβ tracers include flutafuranol, also known as ^[18^F]AZD4694 ([^18^F]NAV4694) (Figure 3) a benzofuran derivative, developed by researchers at AstraZeneca in Sweden [123]. Its development, amongst other second generation of ^18^F-labeled Aβ imaging agents [124] was spurred by the report that flutemetamol and florbetaben, have high level of non-specific white matter retention [116,125,126], which could be a limitation in situations when insoluble Aβ levels are low, due to a spillover effect of radioactivity to adjacent cortical regions from nonspecific binding in white matter. Hence, they may not be useful for correct mapping of Aβ plaque load in low-density regions and in prodromal phases of AD.

Using the intravenous cassette dosing technique to compare the pharmacokinetics of flutafuranol and flutemetamol, it was seen that they were both readily taken up in the brain tissue and washed out of the brain normal rats between 2 and 30 min, but with less than 10% of concentration of flutafuranol at 2 min remaining at 30 min, a time point at which flutemetamol still had up to 28% of the initial concentration at 2 min (Table 1) [103]. With its fast binding kinetics, it could perform better than other Aβ tracers, like PiB, which display, based on time-activity curves, slower kinetics with a blunt peak of specific binding accompanied by a slower decline [95,127]. Consequently, its rapid binding kinetics makes quantification using data based on short acquisition possible. Furthermore, using the cerebellum as a reference region in approaches like reference Logan, valid estimates of Aβ binding could be easily acquired [128]. It is presently in its phase III of clinical trial for the evaluation of its efficacy and safety for the detection of cerebral Aβ in comparison with postmortem histopathology [129,130].

A benzoxazole derivative [^18^F]MK-3328 (Figure 3), which was selected amongst four other fluoroazabenzoxazoles owing to its favorable kinetic profile, shown in rhesus monkey PET studies, a relatively low binding potential in white matter and cortical grey matter, which is approximately 2× lower than that of florbetapir, a relatively lower lipophilicity at log D 2.91, in comparison with an analog [^18^F]AD-269 with similar properties, but more (1.21 fold) lipophilic (Table 1).

In autoradiography studies, it was observed that in an AD patient brain slice that MK-3328 showed punctuate, displaceable binding in the cortical gray matter, with no noticeable binding in the cerebellum [131]. Investigation of the tracer in healthy human volunteers and AD subjects was also being carried out at the time until the premature termination of the clinical trial after the completion of phase 1 of its clinical trial [132].

**Figure 4:**
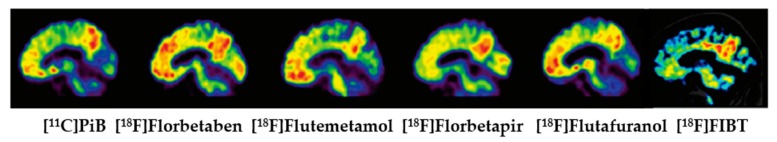
Exemplary sagittal PET images of the FDA approved Aβ PET-tracers of Alzheimer’s disease patients with other select featured tracers, [^11^C]PiB, [^18^F]Florbetaben, [^18^F]Flutemetamol, [^18^F]Florbetapir, [^18^F]Flutafuranol, and [^18^F]FIBT (reproduced with permission as agreed by Newlands Press Ltd. [135]).

The best imidazobenzothiazole derivative [^18^F]FIBT (Figure 3) was reported by the Yousefi et al. group and it was described as the first high-contrast Aβ-imaging agent on par with florbetaben (Figure 4). It also displayed excellent pharmacokinetics, selectivity and high binding affinity to Aβ fibrils in vitro and in vivo comparable to the gold standard PiB [133,134,135]. 

Their results also showed that FIBT has a better pharmacokinetic profile and specific binding affinity to Aβ than florbetaben in transgenic mice. This could be expected from a tracer with >300-fold selectivity for Aβ in comparison to the other amyloid protein aggregates a K_i_ >> 1000 nM to recombinant tau and K_i_ >> 1000 nM to α-syn aggregates [114,136]. Further investigations of the tracer in human subjects are however yet to be carried out [133,135].

#### 2.1.3. The Clinical Utility and Consequences of Clinically Approved PET-Aβ Radiotracers

Since the clinical approval of the abovementioned three FDA approved PET-Aβ tracers as diagnostic tools for the detection of neuritic (Aβ) plaques in live patients, there have been studies to determine their clinical usefulness in the diagnosis AD. These studies have been subsequently and specifically well-reviewed by Kim et al. [137], Barthel et al. [138], Chiotis et al. [139].

In general, the studies have showed that the use of the Aβ-PET tracers led to a moderate to significant change in diagnosis, diagnostic confidence [140,141,142,143,144,145], and had a substantial impact on change in the treatment and management plan of AD [137,144,146]. It is likely that the new generation of Aβ-PET tracers with improved pharmacokinetics will allow for improved signal-to-noise ratio, hence will be more suitable for the quantification of disease progression and therapeutic monitoring.

### 2.2. PET-Tracers for the Imaging of Tau Aggregates

As mentioned earlier, the tau protein plays a key role in the pathogenesis of AD [2,6,11,12]. The predominant aggregation of certain MAPT (tau gene) isoforms, either the 4-repeat (4R tau) or the 3-repeat (3R tau) isoforms have been widely described in tauopathies. So, in addition to the already mentioned properties every CNS tracer should possess [65,67,68,73,74], tau tracers must also address 3R and 4R tau deposits. 3R and 4R tau proteins are the classifications of the 6 tau isoforms according to their tubulin-binding domains [147,148]. In a normal brain, there are equal amounts of the 3R and 4R tau proteins, as well as in AD. An imbalance in tau ratio can lead to abnormal tau accumulation and lead to NDD as in tauopathies. For instance, there is an ample amount 4R tau in PSP, CBD, and argyrophilic grain disease, in contrast, there is an abundance of the 3R tau in Pick’s disease (PiD) [149]. Furthermore, tau tracers should also be able to bind to different tau folds, all of which will facilitate the detection of tau pathology in both AD and non-AD tauopathies [150,151].

The identification of lead compounds for the imaging of Aβ proteinopathies has been relatively easier since most β-sheet binding ligands have a high affinity for Aβ fibrils, with which NFTs coexist in AD and both are colocalized in the gray matter structures [42,152]. In spite of controversy surrounding the subject, PHFs predominantly found in NFTs in in vitro experiments suggest a β-sheet structured core similar to that characteristic of Aβ and α-syn fibrillar aggregates [42]. There are recent reports that there are α-syn containing aggregates present in AD [79,153]. Therefore, tau PET tracers should be selective for tau aggregates over these aggregates as well. 

In AD, there is a distinct difference in the concentration of Aβ relative to tau aggregates. The concentration of Aβ is approximately 5–20 times higher than that of tau aggregates [154]. In spite of this inequality in quantity, there is however a clear-cut regional pattern of Aβ and tau deposition in the neocortex. The frontal cortex has the highest concentration of Aβ aggregates, while the temporoparietal cortices have the highest concentrations of tau aggregates. Different distributions of tau aggregates are also found in the different phenotypes. Although this has its own merits as it will facilitate differential diagnosis of tauopathies, it means that it is unlikely a single tau PET tracer could bind to the whole spectrum of tau polymorphism [42]. In this review, some select selective tau tracers already evaluated in human subjects will be examined, together with other notable tau tracers.

#### 2.2.1. First Generation of Tau-PET Tracers

##### The Arylquinolines

**Figure 5:**
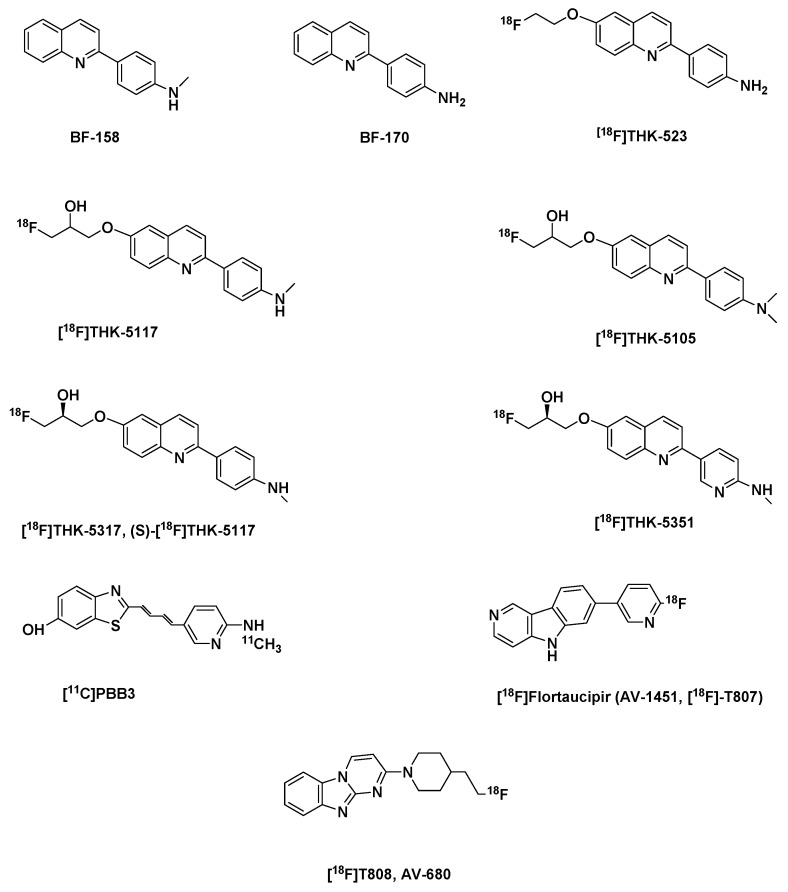
Structures of the first generation tau-PET tracers: BF-158, BF-170, [^18^F]THK-523, [^18^F]THK-5105, [^18^F]THK-5117, [^18^F]THK-5317(17), (S)-[^18^F]THK-5117 ([^18^F]THK-5351), [^11^C]PBB3, [^18^F]Flortaucipir (AV-1451, [^18^F]T807), [^18^F]T808.

The THK-compounds (Figure 5) were as a result of the structural modification of the lead compounds BF-158 and BF-170, arylquinoline derivatives. Even though in vitro fluorescence binding affinity assay data and neuropathological suggested that they are good tau ligands, they showed poor selectivity over amyloid plaques, and furthermore were unable to bind to tau present in non-AD tauopathies. However, autoradiographic studies in AD brain section showed an uptake BF-158 in brain regions which were NFT-rich. Biodistribution studies, analyzed using HPLC with a fluorescence detector, showed a good uptake BF-158 (11.3% ID/g at 2 min p.i.) of BF-158 in the brain of normal mice but a slow washout with only 27.4% of the concentration at 2 min washed out at 30 min, which suggested a high unspecific binding (Table 2). In contrast BF-170 performed better in the biodistribution studies with good brain uptake, as well as a faster washout at 30 min than BF-158 [155] (Table 2).

Structural modification of BF-170 led to the development of [^18^F]THK-523 (Figure 5) a [^18^F]fluoroethoxy derivative. The introduction of an alkylether in the C6 position of the arylquinoline structure improved its affinity and selectivity for tau aggregates relative to BF-170 [156]. However, competition studies showed that it has a relatively low affinity for recombinant tau fibrils (K_i_ 59.3 nM) and even lower for PHF in AD brain homogenates (K_d_ 86.5 nM) than synthetic heparin-induced tau polymers (HITP) K_d_ 1.67 nM (Table 2), an evidence of the inadequacies of synthetic tau preparations, which fails to completely replicate native tau aggregates in vivo [156,157]. 

Notwithstanding, it performed better in vivo than it did in vitro. In comparison to healthy controls, it showed higher cortical retention in AD subjects and was distributed in the brain in accordance with reported histopathological brain distribution of PHF in AD. Unfortunately, due to its high white matter retention, a clear visualization of PET scans was not possible, and for this reason, it was not further developed [158].

Introduction of a secondary alcohol in the fluoroethoxy chain in BF-170 and the monomethylation [^18^F]THK-5117 and dimethylation [^18^F]THK-5105 (Figure 5) of the aniline moiety gave tracers with higher in vitro affinities (K_d_) for both synthetic HITP tau fibrils and for human AD-PHF tau aggregates than [^18^F]THK-523: they showed a 16-fold and 32-fold increase in affinity for human AD-PHF tau aggregates in comparison to their direct predecessor [^18^F]THK-523, a higher in vitro selectivity for tau versus Aβ; a coincidence with Gallyas-Braak staining and immunoreactive tau staining in autoradiography staining of human AD brain sections but not with the distribution of PiB and an good initial brain uptake and washout in normal mice than [^18^F]THK-523 [157] (Table 2).

An improved selectivity could be due to the secondary alcohol present, a polar terminus in the molecules. Likewise the presence of a secondary amine in [^18^F]THK-5117 and a tertiary amine in [^18^F]THK-5105 seemed to be behind the enhancement of tau affinity and better pharmacokinetic profile [35].

However, the N-dimethylation of [^18^F]THK-5105 appeared to be its undoing: due to its relatively higher lipophilicity (1.3x) than [^18^F]THK-5117 (Table 2), it showed in vivo nonspecific binding in the brainstem, thalamus and subcortical white matter, which hinders interpretation. Notwithstanding, it was still able to differentiate between AD patients and healthy control in its first-in-human PET studies. Its distribution in the mesial and lateral lobes of AD patients is in accordance with the reported NFT distribution in AD brain. Nevertheless, due to its inadequacies in comparison to other known tau-PET tracers, it was not used further [159,160].

On the other hand, first human PET studies with [^18^F]THK-5117, demonstrated that it has faster kinetics and better signal to noise ratio than seen in [^18^F]THK-5105, in comparison to which it is less lipophilic. Clinical studies have been conducted with the (*S*)-enantiomer [^18^F]THK-5317, owing to its better signal-to-noise ratio and pharmacokinetics than the (*R*)-enantiomer, a trait observed in the quinoline derivatives [159,161,162,163]. One of the shortcomings of [^18^F]THK-5117 and its S-enantiomer [^18^F]THK-5317 is their significant white matter binding, which might be owing to binding to β-sheet structures of myelin basic protein [164].

In order to reduce white matter binding a feature common among ^18^F-labeled amyloid tracers, a structural modified (S)-[^18^F]THK-5117 was developed, the phenyl ring was replaced with a pyridinyl ring, which made [^18^F]THK-5351 more hydrophilic [164]. It not only displayed a quicker white matter washout (lower white matter retention) and higher specific binding to AD tau-associated regions of interest than [^18^F]THK-5317, but also its retention correlated with extra-hippocampal sub-regional atrophy rather than hippocampal subfields, proffering hence different underlying mechanisms of atrophy in early AD. Another remarkable advantage it has over other tau tracers was the lack of significant retention in the choroid plexus or venous sinus, which could probably lead to a spill-in of tracer signals into the brain [165,166].

Unfortunately, it has been reported to have high affinity to monoamine oxidase-B (MAO-B) (an isoform of monoamine oxidase whose function is to catalyze the oxidation of monoamines [167,168]) in contrast with [^18^F]THK-5117, and also showed a greater off-target binding in the midbrain, thalamus and the basal ganglia [169,170].

##### The Phenylbutadienylbenzothiazoles (PBB)

Following the observation that ligands with a π-electron-conjugated backbone longer than 13Å showed affinities for pathological inclusions in a several tauopathies Maruyama et al. investigated the affinities of a series of compounds with a different structural dimension to tau aggregates and concluded that a core structure with specific distance from 13–19 Å contributes to affinity for non-AD inclusions. Additionally, since ligands with a slender and flat backbone have the ability to transverse and attach to channel-like channels in β-pleated sheets they developed a class of compounds phenyl/pyridinyl-butadienyl-benzothiazoles/benzothiazoliums (PBBs) [171]. They are structural analogs of fluorescent amyloid dye Th-T, with an all-trans butadiene bridge between the aniline and benzothiazolium moieties. Interestingly the resulting tracers were also able to detect tau inclusions in non-AD tauopathies like CBD, Pick’s disease and PSP [172].

Amongst a series of analogs, [^11^C]PBB3 (Figure 5) was selected as the best candidate with an affinity K_d_ for HITP 2.55 nM and nearly 50 folds selectivity for tau versus Aβ fibrils [35,172] (Table 2). Based on preclinical findings in mice, the tracer was further evaluated in humans. It showed in comparison with control accumulation in the medial temporal region of AD subjects. Its distribution in AD human brains differed from that of PiB, suggesting minimal nonspecific binding to white matter, although in both controls and AD brains it accumulated in dural venous sinuses. The use of the tracer in a CBD patient showed its retention in the basal ganglia, hinting that it could be useful for the imaging non-AD tauopathies additionally [172].

**Figure 6:**
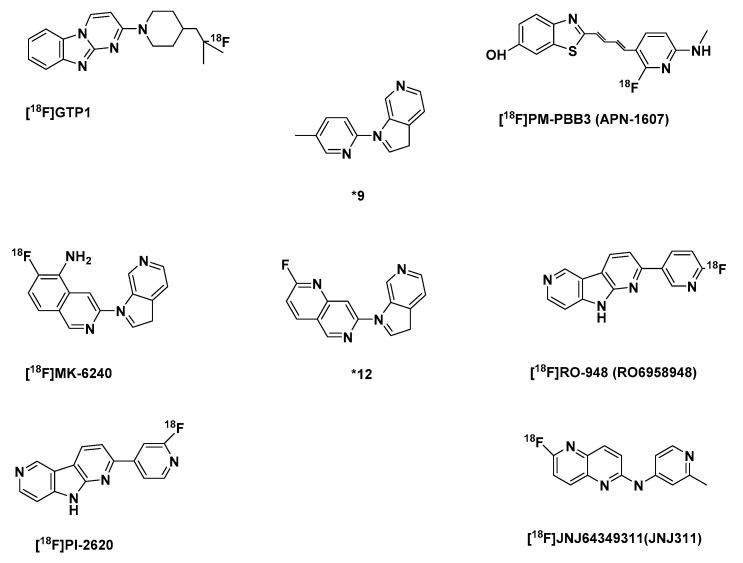
Structures of the selected second-generation tau-PET tracers. [^18^F]GTP1, [^18^F]PM-PBB3 (APN-1607), *9, [^18^F]MK-6240, *12, [^18^F]RO-948 (RO6958948), [^18^F]PI-2620, [^18^F]JNJ64349311(JNJ311).

The compound although it seemed to be a likely candidate for the in vivo imaging of tau pathology, regrettably had some in vitro and in vivo instability problems. The in vitro instability was due to its photoisomerization tendencies: the quick interconversion of E/Z isomers in the presence of light. Although, this can be suppressed by shielding it from light during radio- and chemical synthesis it still is an inconvenience. In vivo, it gets quickly metabolized in mice and humans, with 2% remaining unchanged in mice at 1 min p.i. and 8% at 3 min p.i. in humans. Although this radiometabolite is polar, in mice it still made image analysis difficult [173]. It also displayed off-target binding in the basal ganglia, the choroid plexus and the longitudinal sinus [172]. Nevertheless, a new fluorinated PBB compound [^18^F]PM-PBB3 (Figure 6) has been developed and is being clinically investigated to find out if there would be an improvement in the shortcomings of [^11^C]PBB3 [51].

Interestingly, in in vitro fluorescent study using postmortem DLB and MSA brain sections PBB3 was colocalized on α-syn in LBs, LNs, and GCIs. In contrast, autoradiographic labeling with [^11^C]PBB3 at 10 nM only showed significant binding in MSA cases in regions with a high density of GCIs in the absence of tau or iron deposits. Since the maximum concentration of [^11^C]PBB3 in human PET scans is roughly 10 nM as presented by Koga et al., it means that α-syn is only detectable by [^11^C]PBB3 in MSA patients with a high density of GCIs [174]. A later in vivo human PET study on MSA patients by Perez-Soriano et al. was consistent with the work carried out by Koga et al., that [^11^C]PBB3 binds to α-syn [175,176].

##### The Carbazole and Benzimidazole Derivatives

A screening campaign at Siemens MI Biomarker Research led to the discovery of these classes of lead series [177]. Further optimization led to the development of flortaucipir (AV-1451, [^18^F]T807) and [^18^F]T808 (AV-680) (Figure 5). They both have sufficient affinity for tau (AD-PHF) 14.6 nM and 22nM based on a Scatchard analysis of autoradiography staining of human PHF-AD brain sections, with K_d(Aβ)_/K_d(tau)_ 25 and 27 respectively, meaning a higher selectivity of tau aggregates over Aβ fibrils (Table 2). The binding of [^18^F]T808 to only one type of binding site of the tau aggregates as seen from the degree of linearity in its Scatchard plot further confirmed its selectivity for tau aggregates over Aβ. Most importantly, they have minimal white matter binding and a good pharmacokinetic profile (Table 2) [178,179].

Initial PET scans of flortaucipir in controls and subjects with AD and mild cognitive impairment demonstrated an accumulation of the tracer with a distinct increasing neocortical distribution in tandem with the severity of dementia [180] according to the known mode of spread of PHF in the brain in agreement with Braak’s staging [181]. In other tauopathies such as PSP and CBD, it was shown in a head-to-head comparison of [^11^C]PBB3 and flortaucipir, that the former binds more avidly to neuronal and glial tau lesions relative to the vague binding of flortaucipir [182]. Similar to [^11^C]PBB3, it is suspected that flortaucipir significantly binds to α-syn in the posterior putamen MSA patients. This, however, is not consistent with in vitro autoradiography results, which so far has proven otherwise [183,184].

First-in-human PET studies with [^18^F]T808 showed similar results as flortaucipir, but with more rapid kinetics. As early as 30 min p.i. [^18^F]T808 images stabilized, but flortaucipir SUVR values after 80 min still fluctuated. Notwithstanding, flortaucipir was selected over [^18^F]T808 for clinical development, because of the metabolic defluorination observed in some cases, and the significant accumulation of fluorine-18 in the skull especially in late time points, that could confound PET images. This prevented further in vivo use of the tracer [185].

Off-target binding has been seen in flortaucipir PET studies in the meninges, striatum, choroid plexus and midbrain. In the analysis of autopsy brain samples, it was found out that flortaucipir also binds to vessels, iron-associated regions, substantia nigra, the leptomeningeal melanin and calcifications in the choroid plexus [186]. Another important off-target of flortaucipir is to both isoforms of the MAO enzyme [167,168,187,188]. Furthermore, there was difficulty in quantification due to the fact that it does not reach a steady-state during a typical imaging duration [189,190].

#### 2.2.2. Second Generation of Selective Tau Tracers

Even though several goals were achieved with the first-generation selective tau tracers like improvement in affinity to both 3R and 4R tau deposits, selectivity of the tracers to tau aggregates versus Aβ plaques, and pharmacokinetics, there remains still the problem of lack of selectivity over other protein aggregates, and brain contents: subcortical white matter accumulation, in conjunction with off-target binding especially to MAO-B enzyme in the basal ganglia. Findings in which the THK-radiotracers and flortaucipir have been implicated following in vitro assessments [169,170,184,188]. Clinical validity could be limited in tauopathies where the accumulation of tau is expected in regions with a high concentration of MAO-B, like in PSP and CBS.

There has, furthermore, also been mounting evidence that the binding of certain tracers such as flortaucipir and ^18^F-labeled THK tracers is not only limited to tau deposits but to other protein deposits, like TDP-43 (transactive response DNA binding protein 43 kDa) predominantly present in patients with semantic dementia. Flortaucipir as well as [^11^C]PBB3 showed in vivo binding in patients expected to have α-synuclein deposits [174,175,176,183,184].

Consequently, efforts are being made by various pharmaceutical companies and research institutes to optimize the binding selectivity and enhance the pharmacokinetic profile of tau PET tracers. Hence, more focus will be paid in this section to improvements in the pharmacokinetic profile and specificity of the new tracers in comparison to their predecessors.

##### Optimized First Generation Tau Tracers

As mentioned earlier [185], [^18^F]T808 had a propensity to metabolic defluorination, which led to the selection of flortaucipir (AV-1451, [^18^F]T807) over it, despite its faster kinetics (Table 2). For this reason, it was deuterated to improve its in vivo stability to defluorination, which resulted in the development of [^18^F]GTP1 (Figure 5). This modification prevented the accumulation of free ^18^F-flouride in the skull in clinical PET study, in which it also distinctly differentiated AD subjects from healthy controls [189,192,193].

In addition to its low nanomolar affinity to tau aggregates and excellent selectivity to Aβ plaque (Table 3), it was reported to bind to non-AD tau aggregates. It also showed no off-target binding especially to MAO-B. Both preclinical and clinical in vivo kinetic studies showed that the tracer has a good pharmacokinetic profile which allows imaging some minutes earlier than flortaucipir [190,194]. Further investigations however still need to be carried out to properly compare these two tracers [193].

An introduction of fluorine-18 in the structure of the first-generation tracer [^11^C]PBB3 gave rise to [^18^F]PM-PBB3 (APN-1607) (Figure 6). In human subjects, it showed in less than 5 min a peak ~2.5 [^18^F]PM-PBB3 SUV in the brain. It has less off-target signals in the basal ganglia than [^11^C]PBB3, and a greater signal-to-background ratio. It showed no significant off-target binding in the basal ganglia and thalamus. Furthermore, it did not show radiometabolites in the brain as did its predecessor [^11^C]PBB3 [192,195]. Phase 0 of its clinical evaluation was completed not so long ago in 2018 [196]. 

A structurally modified version of flortaucipir whose inadequacies were already discussed [186,187,197], [^18^F]RO-948 (RO69558948) (Figure 6) was developed and selected from three lead compounds. Of the selected three which also displayed good brain uptake, fast brain clearance, high affinity for NFT (Table 3) and excellent selectivity against Aβ plaques in AD brain tissue, lower affinity for MAO-A and MAO-B in comparison to [^18^F]T807 and [^18^F]THK-5351, and based on preclinical binding study RO-948 was selected for further development. This was because in comparison to the other analogs it displayed better pharmacokinetics and metabolic properties both in mice and non-human primates. Moreover, it showed a better signal-to-background ratio than the others in AD patients. Notwithstanding, three of them gave results from their first-in-human study, which were consistent with preclinical data [198,199,200].

Upon the discovery that affinity for MAO-A is significantly attenuated and high affinity for aggregated tau improved in the presence of pyrrolo[2,3-b:4,5-c’]dipyridine core structures in comparison to pyrido[4,3-b]indole core structure a series of fluoropyridine regioisomers were developed from which the 4-pyridine regioisomer [^18^F]PI-2620 (Figure 6) a regioisomer of RO-948 was selected. In AD brain homogenate competition assays, it demonstrated a high affinity for tau deposits pIC50 8.4 nM (Table 3), and a superior binding to both 3R and 4R tau aggregate folds in self-competition experiments using recombinant K18 fibrils (representing 4R tau pathology) as well as human PSP and PiD brain homogenates. 

Besides, it is selective over Aβ and has no off-target binding towards either MAO-A as [^18^F]RO-948 or MAO-B as flortaucipir, and furthermore showed low off binding in brains of non-demented controls, with rapid and complete washout. It also showed selective binding to pathological tau present in Braak I, III and V human brain sections in autoradiography experiments. In autoradiography studies it also showed to tau aggregates/folds in PSP brain sections, which of course has been controversial, since many tau tracers has been reported not to be bind to tau deposits in PSP in autoradiography experiments. However, off-target binding was observed in the pars compacta portion of the substantia nigra in human brain sections, consistent with the affinity of some tau tracers like flortaucipir, [^18^F]MK-6240 to melanin-containing cells [52,53,201].

Clinical data are needed to confirm the usefulness of [^18^F]PI-2620 in non-AD patients. Nonetheless, it is presently being examined in several clinical trials in order to establish its pharmacokinetic profile in humans, and decide its application in in vivo PET-imaging of tau aggregates/folds both in non-AD and AD tauopathies [53].

##### The Azaindole-Isoquinoline and Naphthyridine Derivatives

Following an SAR study, an azaindole-isoquinoline derivative was developed. The study showed that the azaindole core (*9) (Figure 6) with a 2,4-substituted pyridine shown below was the minimum pharmacophore needed for a high binding affinity to NFTs. The insertion of fluorine in the minimal pharmacophore led to a loss in affinity by >10 fold. This was also observed when either pyridinyl rings were fluorinated. A phenomenon, which hinted at a specific electronic contribution of the basic nitrogen to NFT binding. In [^18^F]MK-6240 there is a minimum effect of fluorine on the basicity of the heterocyclic nitrogen in the isoquinoline ring and the presence of a primary amine, an additional stronger basic center, must have improved its affinity to NFTs, K_d_ 0.36, in comparison to the 1,6-naphthyridine derivative (*12), with both basic centers in the ring, with affinity to NFTs, K_d_ 52.6 [202] (Table 3)

It exhibited favorable pharmacokinetics, with a fast brain uptake and clearance (Table 3). Uptake was higher in AD subjects and was considerably higher in brain regions expected to have NFT like in the hippocampus, but very low uptake in the cerebellar gray matter suggests a potential use of the cerebellar gray matter as a reference region. Based on reliability analysis simplified quantitative approaches could offer informed estimates of NFT load [203]. Furthermore, the spatial patterns of binding of the tracer were in accordance with the neuropathological staging of NFT, as reported from recent clinical studies [204].

Preclinical findings confirmed a lack of binding to MAO-A and MAO-B [203]. Unlike flortaucipir and [^18^F]THK-5351 off-target binding was not seen in the choroid plexus and basal ganglia [204], but like flortaucipir and various tau PET tracers, off-target binding to neuromelanin- and melanin containing cells like the pigmented neurons in the substantia nigra, and meninges was observed [52,187,201]. To confirm initial observations, there are ongoing clinical trials on non-AD patients. The phase I of its clinical trial was completed in 2016 [205]. 

A 1,5-napthyridine derivative, [^18^F]JNJ64349311(JNJ311) (Figure 6) was also reported. It showed moderate initial brain uptake but a fast brain clearance in biodistribution assay using wild-type mice (Table 3). It is quickly metabolized as was seen in NMRI mice and a rhesus monkey 30 min after intravenous injection, where only 22% and 35% respectively of the recovered activity was the intact radioligand. However, all the detected radiometabolites were more polar than the tracer, and none was found in the brain even at 60 min after injection. Furthermore, no bone uptake was detected in a rhesus monkey during a 120 min scan, in the duration of a microPET scan, which also showed a moderate initial brain uptake (SUV of 1.9 at 1 min p.i.) with a rapid wash-out.

Semi-quantitative autoradiography studies on post-mortem tissue sections of human AD brains displayed highly displaceable binding to NFT-rich regions, but it showed no specific binding to human PSP and CBD brain slices. Based on its in vitro and in vivo preclinical profiling, it was deemed a promising candidate for quantitative tau PET imaging in AD [206]. There is presently no in vivo human data for the tracers of the JNJ series.

### 2.3. Selective PET-Tracers for the Imaging of α-syn

Most of the PET/SPECT tracers developed and approved so far for the differential differential diagnosis of PD have been geared towards the evaluation of the function of the dopaminergic system [207]. As was mentioned earlier, 50% of substantia nigra cells (stage III of the Braak staging) and a probable loss of a higher percentage of dopaminergic nerve endings in the putamen have to be lost before the appearance of motor symptoms [71,72,73], in contrast, based on the findings of Braak et al., there is deposition of α-syn in LBs and LNs, which occurs sequentially and additively throughout the VI stages of disease progression [74], therefore the most accurate and earliest detection of premotor PD should be based on imaging α-syn instead of dopaminergic changes [208].

The development of α-syn PET tracers is still an unmet need and in its early stages. Regardless, efforts have been made in the past decades and are still being presently made to develop tracers with a high affinity and selectivity for α-syn over Aβ and tau aggregates.

#### 2.3.1. The Phenothiazine Derivatives

**Figure 7:**
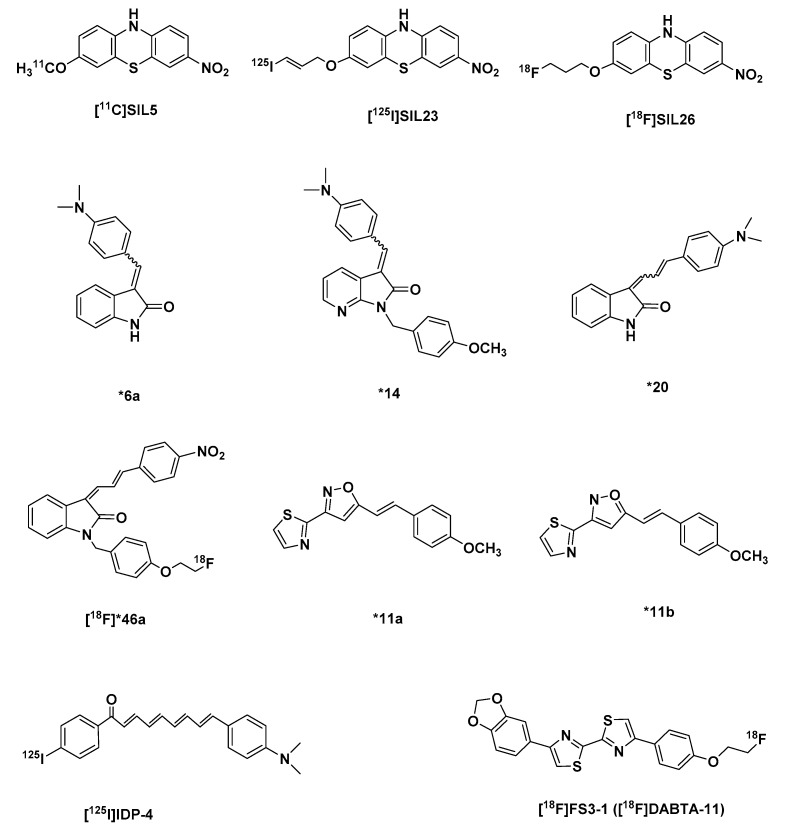
Structures of selective α-syn-PET tracers: [^11^C]SIL5, [^125^I]SIL23, [^18^F]SIL26, *6a, *14, *20, *46a, *11a, *11b, [^125^I]IDP-4, [^18^F]FS3-1 ([^18^F]DABTA-11). *numbers given to the tracers in their respective publications.

In 2011, to discover selective α-syn tracers Yu et al. synthesized a series of phenothiazine derivatives. Three of the tricyclic compounds (Figure 7) based on in vitro Th-T competition assay to recombinant α-syn fibrils were selected: [^11^C]SIL5, [^125^I]SIL23, and [^18^F]SIL26 based on the fact that they displayed an affinity (K_i_) to the α-syn fibrils less than 60 nM [209] (Table 4).

In further tests, [^125^I]SIL23 with a k_i_ of 57.9 nM [209] was able to bind to α-syn fibrils in postmortem PD brain homogenates, which indicated that the tracer binding affinity in PD brain samples is comparable to its affinity to recombinant α-syn fibrils. It also displayed 5-fold and 2-fold less affinity for Aβ_1-42_ and tau aggregates respectively in comparison to α-syn fibrils, however this selectivity was insufficient for in vivo imaging. Moreover, a high nonspecific binding in white matter seems to limit autoradiography with the tracer initial experiments. Furthermore, its affinity for α-syn fibrils K_d_ 148 nM, is also not optimal for the in vivo imaging of α-syn fibrils [210].

[^11^C]SIL5 and [^18^F]SIL26 with binding affinities (K_i_) 1.8× and 1.2× more than that of [^125^I]SIL23 respectively, showed a low initial brain uptake in healthy Sprague-Dawley rats 5 min p.i. 0.953%ID/g and 0.758%ID/g respectively, and slow washout with [^18^F]SIL26 performing poorer of the 2 tracers with not less than 50% of the initial brain concentration at 5 min remaining at 60 min. [^11^C]SIL5 still had at 60 min 16.57% of its initial brain uptake at 2 min, which suggested a slow washout of the tracer or a lot of unspecific binding (Table 4).

In vivo microPET imaging in a healthy cynomolgus macaque confirmed that [^11^C]SIL5, with a faster washout kinetics of the two was able to penetrate the BBB into the brain, and also has a homogeneous distribution and fast washout kinetics. The authors believe that both compounds require further structural optimization in order to make them a more suitable α-syn tracer [211].

#### 2.3.2. The Indolinone and Indolinonediene Derivatives

An SAR study by structural modifications of an indolinone derivative *6a [212] (Figure 7), by the introduction of different alkyl and arylalkyl groups substituted at the indolinone nitrogen led to the identification of an aza-analog-*14 (Figure 7), which was the most successful in this series (Table 3). With a binding affinity K_i_ 79 nM to recombinant α-syn fibrils less than that of the select 3 phenothiazine tracers [209,213], with 1,4- and 11-fold selectivity over recombinant Aβ and tau aggregates. Another major limitation of the series was the presence of E/Z isomers, which re-equilibrate after separation.

A homologation of the double bond in *6a by the addition of an additional double (to form diene group) bond, in order to increase affinity to α-syn fibrils over the other aggregates gave a series of compounds, which were a mixture of stereoisomers with either an E,E or Z,E configuration, which quickly re-equilibrated after chromatographic purification. *20 (Figure 7) was however selected from the series based on its improved affinity (K_i_) for recombinant α-syn fibrils 1.9× more than *14, but unfortunately affinity K_i_ 27.6 nM for Aβ increased as well, with 1.3-fold selectivity over tau aggregates (Table 4). However, its strong fluorescent attributes allowed for a performance of fluorescent microscopy studies of postmortem AD and PD brain samples. Results showed that it labels both LB and Aβ plaques. Regardless, this showed that indolinonediene derivatives can label α-syn fibrils in LBs.

Introduction of a para nitro group into the pendant benzene ring of the diene moiety made it possible to isolate both the *E,E* and *Z,E* stereoisomers, and further explore their in vitro properties. The *Z,E* regioisomers were generally more active than the corresponding *E,E* configuration in terms of higher affinity for α-syn over Aβ and tau fibrils. The best in this series was [^18^F]46a with the highest affinity (K_i_) for α-syn fibrils 2 nM, 70-fold and 40-fold less affinity for Aβ and tau fibrils respectively (Table 4).

Unfortunately, due to its high lipophilicity log D 4.18, which prevented obtaining a reliable and reproducible results from binding assays to insoluble α-syn acquired from PD brain and the possible reduction of the nitro group to an amino group in vivo makes it an unsuitable PET probe for imaging LB and LN in PD subjects. In spite of the shortcomings of the compound it showed interesting selectivity for α-syn fibrils over Aβ and tau, and could serve as a good lead for further development of α-syn fibril tracers [213].

#### 2.3.3. Chalcone Derivatives and Structural Cogeners

Hsieh et al. went further to investigate a series of chalcone derivatives, whose enone moiety serves as an isosteric replacement of the diene group in the indolinonediene derivatives while precluding the *E,E* and *Z,E* isomerization problem. The indole ring was further replaced with a benzothiazole ring system, based on a previous SAR study, which revealed that an electron-deficient ring like the aza-indole system has a higher affinity for α-syn fibrils relative to the indole ring system and to prevent the Michael-acceptor properties associated with the chalcone system they replaced the enone moiety with an isoxazole and a pyrazole ring system. The results of a competition in vitro binding studies with Th-T led to the identification of a compound *11a,b (37 a,b) (Figure 7), isoxazole derivatives with a modest affinity in comparison to [^18^F]46a (36) for α-syn at K_i_ 18.5 nM over Aβ and tau fibrils with 5-fold and over 54-fold less affinity respectively (Table 4). Although the compounds described in their report have modest affinity to serve as a PET radiotracer for in vivo imaging studies, they could, however could be used for further SAR studies [214].

Based on previous research, it was discovered that flavonoids could inhibit not only the formation of Aβ [215,216,217] but also of α-syn [218,219,220] aggregates, an indication that they could also bind with α-syn aggregates. With this in mind, Ono et al. developed some α-syn imaging tracers based on the chalcone scaffold: they developed four prospective chalcone derivatives (IDP compounds) with varying molecular lengths made possible by conjugated double bonds. A longer molecular length and a long conjugated π system were believed to lead to increased affinity of probes to Aβ [221], and tau aggregates as was seen in the PBB compounds [172,222].

All the IDP-compounds, unfortunately, displayed almost as much affinity for Aβ as they displayed for α-syn (Table 4), with affinity for α-syn increasing proportionately to molecular length, with not really much change in the selectivity to Aβ, which means that [^125^I]IDP-4 with a tetraene structure was the best in this regard. It had a high binding affinity to α-syn K_d_ of 5.4 nM and 3-fold selectivity to recombinant Aβ fibrils, which nevertheless was not as high as [^18^F]46a, which the authors believe could be attributed to different binding assay conditions.

[^125^I]IDP-4 (Figure 7) in vivo biodistribution in normal mice performed poorer than the other IDP-compounds, with a brain uptake of 0.45%ID/g at 2 min p.i. and a low brain clearance, 93.3% of the concentration at 2 min still remaining at 60 min (Table 4). The suboptimal pharmacokinetics could be due to the lipophilicity, high molecular weight and chemical structure of the tracer. In any case, this property makes it an unlikely tracer for in vivo imaging of α-syn aggregates. Still, it could be a useful probe for in vitro screening of compounds for affinity to α-syn and Aβ fibrils in vitro [222].

#### 2.3.4. Diarybisthiazole Compounds

More recently, highly innovative small molecules utilized as diagnostic probes, based on 4,4’-diaryl-2,2’-bithiazole, called DABTAs were developed by Yousefi et al. as sensitive, selective and specific tracers. According to pilot data these compounds suitable for the visualization and quantification of α-syn pathology by nuclear medicine imaging. With the help of these newly developed ligands, the presence, distribution and progression pattern of α-syn can be investigated in the living brain, reflecting the disease entity and stage. Preliminary results with the [^18^F]FS3-1 ([^18^F]DABTA-11) (Figure 7) in a rat model overexpressing human E46K mutated α-syn are promising regarding sensitivity [223,224]. In addition, in vivo imaging of the progression and spreading of α-synuclein pathology over time with the tracer greatly motivates the authors to develop tracers for aggregated α-syn in PD, DLB or MSA.

## 3. Conclusions

Several Aβ PET tracers have been developed and promoted discussion on their clinical values. Among these three tracers have been already approved by the FDA and EMA. Research on tau PET yielded several tau-tracers already entered into clinical investigations, however, still inherent are challenges in selective tau imaging. The lack of any consented and approved tracer for aggregated α-syn greatly motivated scientists in the field to develop tracer for aggregated α-syn in PD, DLB or MSA. Remarkable progress has been made so far in order to fulfill the unmet need for α-syn PET tracers with suitable pharmacokinetics, binding affinity and selectivity. Lately, innovative small molecules have been identified with excellent binding affinity and selectivity for α-syn fibrils relative to Aβ and tau aggregates. Utilizing high-throughput binding assays, in-silico design in the conventional tracer development may accelerate the identification of new leads for a specific α-syn PET imaging.

## Figures and Tables

**Table 1:** Binding affinities and Pharmacokinetics of featured Aβ PET-tracers.

Tracer	Log P	Aβ_(1-40)_ fibrils, [nM]		Aβ_(1-42)_ Fibrils, [nM]		Aβ plaques in Brain Homogenates, [nM]		Brain Uptake [%ID/g] (2 min p.i.)	Brain Clearance [%ID/g] (30 min p.i.)
		K_i_	K_d_	K_i_	K_d_	K_i_	K_d_		
Th-T [92,96]	0.57	890 580	NA	NA	NA	NA	NA	NA	NA
[^11^C]PiB [91,94] [105,131]	1.2 2.23	4.3	4.7	NA	NA	IC_50_: 2.3	1.4	0.21%ID-kg/g ^1^ 1.50 (5 min p.i.)	0.018%ID-kg/g ^1^ 0.31
[^18^F]Florbetaben [114,119]	2.41	NA	NA	NA	NA	6.7 2.22	NA	7.77	1.59
[^18^F]Florbetapir [119]	NA	NA	NA	NA	NA	2.87	3.72	7.33	1.88 (60 min p.i.)
[^18^F]Flutemetamol [103,105]	3.2 ^2^	15.3	1.6	NA	NA	NA	NA	3505 nM	980 nM
	NA	NA	NA	NA	NA	NA	NA	3.67 (5 min p.i.)	0.42
[^11^C]6-Me-BTA-1 [92,94,96]	3.36	20.2 10	NA	NA	NA	NA	NA	7.61 0.223%ID-kg/g ^1^	2.76 0.083%ID-kg/g ^1^
[^11^C]SB-13 [110,112]	2.36	6.0	NA	NA	NA	1.2	NA	1.15 (cortex) 1.15 (cerebellum)	0.42 (cortex) 0.41 (cerebellum)
[^18^F]FMAPO [112,114]	2.95	NA	NA	NA	NA	5.0	NA	9.75	1.70
[^18^F]Flutafuranol [^18^F]AZD4694 [103]	2.8 ^2^	18.5	2.3	NA	NA	NA	NA	1550 nM	154 nM
[^18^F]MK-3328 [131]	2.91	NA	NA	NA	NA	IC_50_: 10.5	NA	NA	NA
[^18^F]AD-269 [131]	3.42	NA	NA	NA	NA	IC_50_: 8.0	NA	NA	NA
[^18^F]FIBT [133,134]	1.92	2.1	NA	NA	NA	NA	0.7	~7.3 ^3^	~1.25 ^3^

The log P values are the partition coefficient (octanol/water) or log D partition coefficient (octanol/PBS) reported in the respective publications.^1^ The samples were weighed to determine the percent injected dose per gram tissue (% ID/g), and this value was multiplied by the whole-body weight (in kg) to determine body-weight normalized radioactivity concentration [(% ID-kg/g)] values. ^2^ ElogD_oct_. ^3^ The values were estimated from bar-charts presented in the publication. NA: data not available.

**Table 2:** Binding affinities and Pharmacokinetics of featured first-generation tau PET-tracers.

Tracer	Log P	Tau Affinity [nM]		Selectivity tau/Aβ	Aβ Affinity (nM)	Brain Uptake [%ID/g]	Brain Clearance [%ID/g]
		HITP	AD-PHF, K_d_ [nM]			2 min p.i.	30 min p.i.
BF-158 [155]	1.67	EC_50_: 399	NA	1.60 ^1^	K_i:_ > 5000	11.3	3.1
BF-170 [155]	1.85	EC_50_: 221	NA	3.50 ^1^	K_i:_ > 5000	9.1	0.25
[^18^F]THK-523 [156,157]	2.40	K_d1_:1.67 K_d2_:21.74 K_i_: 59.30	86.50	10 ^2^	K_d1_ (Aβ fibrils): 20.7	2.75	1.47
[^18^F]THK-5105 [157]	3.03	K_d1_:1.45 K_d2_:7.40 K_i_: 7.80	2.63	25 ^2^	K_d1_ (Aβ fibrils): 35.9	9.20	3.61
[^18^F]THK-5117 [157]	2.32	10.50	5.19	30 ^2^	NA	6.06	0.59
[^18^F]THK-5351 [164]	1.5	NA	2.9	NA	NA	NA	NA
[^11^C]PBB3 [172,173]	3.3	NA	K_d_: 2.55 ^3^	48 ^2^	K_d_: 114 ^3^	1.92 (1 min p.i.)	0.11
[^18^F]Flortaucipir (AV-1451,[^18^F]T807) [178,191]	1.67	NA	14.6 ^3^	25 ^2^	NA	4.43 (5 min p.i.)7.5	0.620.8
[^18^F]T808 [179,191]	NA	NA	22	27 ^2^	NA	4.9	0.4

The log P values are the partition coefficient (octanol/water) or log D partition coefficient (octanol/PBS) reported in the respective publications. Selectivity tau vs Aβ: ^1^ EC_50(Aβ)_/EC_50 (tau)_, ^2^ K_d(Aβ)_/K_d(tau)_. NA: data not available. ^3^ Autoradiographic binding to plaque- and tangle-rich regions in AD brains.

**Table 3:** Binding affinities and Pharmacokinetics of featured tau PET-tracers.

Tracer	Log P	Tau affinity [nM]		Selectivity, tau/Aβ	K_i_, Aβ fibrils (nM)	Brain Uptake [%ID/g]	Brain Clearance [%ID/g]
		**HITP**	**AD-PHF**			**2 min p.i.**	**30 min p.i.**
[^18^F]GTP1 [193]	NA	NA	K_d_:10.8	NA	NA	NA	NA
[^18^F]PM-PBB3 (APN-1607)	NA	NA	NA	NA	NA	NA	NA
(*9) [202]	NA	NA	K_i_: 8.8	>1136^1^	>10000	NA	NA
[^18^F]MK-6240 [202]	3.32	NA	K_i_: 0.36	>27777^1^	>10000	NA	NA
(*12) [202]	2.90	NA	K_i_: 52.6	>190	>10000	NA	NA
[^18^F]RO-948 (RO6958948)[53] [199]	3.22	NA	44% ^2^ pIC_50_: ^3^ 8.4	NA	pIC_50_: < 6 ^4^	5.7 ^5^	10.9 ^6^
[^18^F]PI-2620 [53]	NA	NA	pIC_50_: 8.5 ^7^	NA	pIC_50_: < 6 ^4^	5.9 ^5^	16.6 ^6^
[^18^F]JNJ64349311 (JNJ311) [206]	2.2	NA	K_i_: 8 ^8^	>500	>4398 ^9^ IC_50_: < 5 ^9^	1.9 ^10^	0.3 ^10^

The log P values are the partition coefficient (octanol/water) or log D partition coefficient (octanol/PBS) reported in the respective publications. Selectivity tau vs Aβ: ^1^ K_i(Aβ)_/K_i(tau_). ^2^ % inhibition of 10 nM of [^3^H]T808 on fresh frozen human brain sections derived from AD cases. ^3^ Self-competition. ^4^ In competition with [^3^H]PiB. ^5^ Peak uptake (injected dose per gram brain; ID/g); ^6^ ratio of peak uptake divided by peak at 30 min. ^7^ In competition with [^18^F]RO-948. ^8^ [^3^H]AV680 as competitor. ^9^ [^3^H]Florbetapir as competitor. ^10^ Data are expressed as SUV mean. NA: data not available. *numbers given to the tracers in the respective publications.

**Table 4:** Binding affinities and Pharmacokinetics of featured α-syn PET-tracers.

Tracer	Log D	α-syn Affinity [nM]			Aβ Fibrils Affinity [nM]		Tau Fibrils Affinity [nM]		Brain Uptake [%ID/g]	Brain Clearance [%ID/g]
		K_i_		K_d_	K_i_	K_d_	K_i_	K_d_	5 min p.i.	60 min p.i.
		α-syn Fibrils	Human PD Homogenate							
[^11^C]SIL5 [209,210,211]	3.79	32.1. 66.2.	83.1	NA	110	NA	136	NA	0.953	0.158
[^125^I]SIL23 [209,210]	5.72	57.9	NA	148	NA	635	NA	230	NA	NA
[^18^F]SIL26 [209,210,211]	4.02	49.0 15.5.	33.5	NA	103	NA	125	NA	0.758	0.410
*14 [[213]	4.2 ^1^	79.5	NA	NA	113.3	NA	853.5	NA	NA	NA
*20 [213]	3.5 ^2^	40.7	NA	NA	27.6	NA	53.7	NA	NA	NA
[^18^F]46a [213]	4.18	2.1	NA	8.9	142.4	NA	80.1	NA	NA	NA
*11a,b [214]	3.54 ^3^	18.5	NA	NA	91.5	NA	>1000	NA	NA	NA
[^125^I]IDP-4 [222]	NA	NA	NA	5.4	NA	16.24	NA	NA	0.45 (2 min p.i.)	0.42

^1,2 and 3^ Calculated by ChemDraw Ultra 13.0 and Professional 15.1 respectively. * numbers given to the tracers in their respective publications. NA: data not available.

## References

[1] Alzheimer’s Disease International (2015). The Global Impact of Dementia: An Analysis of Prevalence, Incidence, Cost and Trends.

[2] Alzheimer’s Disease International (2018). World Alzheimer Report 2018—The State of the Art of Dementia Research: New Frontiers.

[3] World Health Organisation-Alzheimer’s Disease International (2012). Dementia: A Public Health Priority.

[4] Trevisan K., Cristina-Pereira R., Silva-Amaral D., Aversi-Ferreira T.A. (2019). Theories of Aging and the Prevalence of Alzheimer’s Disease. Biomed. Res. Int..

[5] Xie A., Gao J., Xu L., Meng D. (2014). Shared mechanisms of neurodegeneration in Alzheimer’s disease and Parkinson’s disease. Biomed. Res. Int..

[6] Murphy M.P., LeVine H. (2010). Alzheimer’s disease and the amyloid-beta peptide. J. Alzheimers Dis..

[7] (2018). Alzheimer’s Association 2018 Alzheimer’s disease facts and figures. Alzheimer’s Dement..

[8] Zhang Y., Thompson R., Zhang H., Xu H. (2011). APP processing in Alzheimer’s disease. Mol. Brain.

[9] Gu L., Guo Z. (2013). Alzheimer’s Aβ42 and Aβ40 peptides form interlaced amyloid fibrils. J. Neurochem..

[10] Kayed R., Lasagna-Reeves C.A. (2013). Molecular mechanisms of amyloid oligomers toxicity. J. Alzheimers Dis..

[11] Trojanowski J.Q., Clark C.M., Schmidt M.L., Arnold S.E., Lee V.M. (1997). Strategies for Improving the Postmortem Neuropathological Diagnosis of Alzheimer’s Disease. Neurobiol. Aging.

[12] Dubois B., Hampel H., Feldman H.H., Scheltens P., Aisen P., Andrieu S., Bakardjian H., Benali H., Bertram L., Blennow K. (2016). Preclinical Alzheimer’s disease: Definition, natural history, and diagnostic criteria. Alzheimers Dement..

[13] Delacourte A. (1998). Les diagnostics de la maladie d’Alzheimer. Ann. Biol. Clin. (Paris).

[14] Takizawa C., Thompson P.L., van Walsem A., Faure C., Maier W.C. (2015). Epidemiological and economic burden of Alzheimer’s disease: A systematic literature review of data across Europe and the United States of America. J. Alzheimers Dis..

[15] Johnson K.A., Fox N.C., Sperling R.A., Klunk W.E. (2012). Brain imaging in Alzheimer disease. Cold Spring Harb. Perspect. Med..

[16] Budson A.E., Solomon P.R. (2012). New criteria for Alzheimer disease and mild cognitive impairment: implications for the practicing clinician. Neurologist.

[17] Jack C.R., Bennett D.A., Blennow K., Carrillo M.C., Dunn B., Haeberlein S.B., Holtzman D.M., Jagust W., Jessen F., Karlawish J. (2018). NIA-AA Research Framework: Toward a biological definition of Alzheimer’s disease. Alzheimers Dement..

[18] Bekris L.M., Yu C.-E., Bird T.D., Tsuang D.W. (2010). Genetics of Alzheimer disease. J. Geriatr. Psychiatry Neurol..

[19] Freudenberg-Hua Y., Li W., Davies P. (2018). The Role of Genetics in Advancing Precision Medicine for Alzheimer’s Disease-A Narrative Review. Front. Med. (Lausanne).

[20] Williamson J., Goldman J., Marder K.S. (2009). Genetic aspects of Alzheimer disease. Neurologist.

[21] Isaac M., Vamvakas S., Abadie E., Jonsson B., Gispen C., Pani L. (2011). Qualification opinion of novel methodologies in the predementia stage of Alzheimer’s disease: Cerebro-spinal-fluid related biomarkers for drugs affecting amyloid burden—Regulatory considerations by European Medicines Agency focusing in improving benefit/risk in regulatory trials. Eur. Neuropsychopharmacol..

[22] Blennow K., Hampel H., Zetterberg H. (2014). Biomarkers in amyloid-β immunotherapy trials in Alzheimer’s disease. Neuropsychopharmacology.

[23] Palmqvist S., Zetterberg H., Mattsson N., Johansson P., Minthon L., Blennow K., Olsson M., Hansson O. (2015). Detailed comparison of amyloid PET and CSF biomarkers for identifying early Alzheimer disease. Neurology.

[24] Hampel H., Bürger K., Teipel S.J., Bokde A.L.W., Zetterberg H., Blennow K. (2008). Core candidate neurochemical and imaging biomarkers of Alzheimer’s disease. Alzheimers Dement..

[25] Zetterberg H., Tullhög K., Hansson O., Minthon L., Londos E., Blennow K. (2010). Low incidence of post-lumbar puncture headache in 1,089 consecutive memory clinic patients. Eur. Neurol..

[26] Reiman E.M., Jagust W.J. (2012). Brain imaging in the study of Alzheimer’s disease. Neuroimage.

[27] Fawaz M.V., Brooks A.F., Rodnick M.E., Carpenter G.M., Shao X., Desmond T.J., Sherman P., Quesada C.A., Hockley B.G., Kilbourn M.R. (2014). High Affinity Radiopharmaceuticals Based Upon Lansoprazole for PET Imaging of Aggregated Tau in Alzheimer’s Disease and Progressive Supranuclear Palsy: Synthesis, Preclinical Evaluation, and Lead Selection. ACS Chem. Neurosci..

[28] DeKosky S.T., Marek K. (2003). Looking backward to move forward: early detection of neurodegenerative disorders. Science.

[29] Bailey D.L., Maisey M.N., Townsend D.W., Valk P.E. (2005). Positron Emission Tomography. Basic Sciences.

[30] van der Born D., Pees A., Poot A.J., Orru R.V.A., Windhorst A.D., Vugts D.J. (2017). Fluorine-18 labelled building blocks for PET tracer synthesis. Chem. Soc. Rev..

[31] Filippi L., Chiaravalloti A., Bagni O., Schillaci O. (2018). 18F-labeled radiopharmaceuticals for the molecular neuroimaging of amyloid plaques in Alzheimer’s disease. Am. J. Nucl. Med. Mol. Imaging.

[32] Mathis C.A., Wang Y., Klunk W.E. (2004). Imaging beta-amyloid plaques and neurofibrillary tangles in the aging human brain. Curr. Pharm. Des..

[33] Klunk W.E., Engler H., Nordberg A., Wang Y., Blomqvist G., Holt D.P., Bergström M., Savitcheva I., Huang G.-f., Estrada S. (2004). Imaging brain amyloid in Alzheimer’s disease with Pittsburgh Compound-B. Ann. Neurol..

[34] Jacobson O., Kiesewetter D.O., Chen X. (2015). Fluorine-18 radiochemistry, labeling strategies and synthetic routes. Bioconjug. Chem..

[35] Ariza M., Kolb H.C., Moechars D., Rombouts F., Andrés J.I. (2015). Tau Positron Emission Tomography (PET) Imaging: Past, Present, and Future. J. Med. Chem..

[36] Barthel H., Sabri O. (2011). Florbetaben to trace amyloid-β in the Alzheimer brain by means of PET. J. Alzheimers Dis..

[37] Johnson K.A., Sperling R.A., Gidicsin C.M., Carmasin J.S., Maye J.E., Coleman R.E., Reiman E.M., Sabbagh M.N., Sadowsky C.H., Fleisher A.S. (2013). Florbetapir (F18-AV-45) PET to assess amyloid burden in Alzheimer’s disease dementia, mild cognitive impairment, and normal aging. Alzheimers Dement..

[38] Hatashita S., Yamasaki H., Suzuki Y., Tanaka K., Wakebe D., Hayakawa H. (2014). 18FFlutemetamol amyloid-beta PET imaging compared with 11CPIB across the spectrum of Alzheimer’s disease. Eur. J. Nucl. Med. Mol. Imaging.

[39] Arriagada P.V., Growdon J.H., Hedley-Whyte E.T., Hyman B.T. (1992). Neurofibrillary tangles but not senile plaques parallel duration and severity of Alzheimer’s disease. Neurology.

[40] Maccioni R.B., Farías G., Morales I., Navarrete L. (2010). The revitalized tau hypothesis on Alzheimer’s disease. Arch. Med. Res..

[41] Jack C.R., Knopman D.S., Jagust W.J., Petersen R.C., Weiner M.W., Aisen P.S., Shaw L.M., Vemuri P., Wiste H.J., Weigand S.D. (2013). Tracking pathophysiological processes in Alzheimer’s disease: An updated hypothetical model of dynamic biomarkers. Lancet Neurol..

[42] Villemagne V.L., Furumoto S., Fodero-Tavoletti M., Harada R., Mulligan R.S., Kudo Y., Masters C.L., Yanai K., Rowe C.C., Okamura N. (2012). The challenges of tau imaging. Future Neurol..

[43] Ke Y.D., Suchowerska A.K., van der Hoven J., de Silva D.M., Wu C.W., van Eersel J., Ittner A., Ittner L.M. (2012). Lessons from tau-deficient mice. Int. J. Alzheimers Dis..

[44] Ludolph A.C., Kassubek J., Landwehrmeyer B.G., Mandelkow E., Mandelkow E.-M., Burn D.J., Caparros-Lefebvre D., Frey K.A., de Yebenes J.G., Gasser T. (2009). Tauopathies with parkinsonism: clinical spectrum, neuropathologic basis, biological markers, and treatment options. Eur. J. Neurol..

[45] Medeiros R., Baglietto-Vargas D., LaFerla F.M. (2011). The role of tau in Alzheimer’s disease and related disorders. CNS Neurosci. Ther..

[46] Winners. https://www.eanm.org/congresses-events/awards-grants/winners/.

[47] Vanhaute H., Ceccarini J., Michiels L., Koole M., Emsell L., Lemmens R., Vandenbulcke M., van Laere K. (2019). PET-MR Imaging of Tau and Synaptic Density in Prodromal Alzheimer’s Disease.

[48] U.S. Food and Drug Administration. https://www.fda.gov/.

[49] Sperling R.A., Aisen P.S., Beckett L.A., Bennett D.A., Craft S., Fagan A.M., Iwatsubo T., Jack C.R., Kaye J., Montine T.J. (2011). Toward defining the preclinical stages of Alzheimer’s disease: recommendations from the National Institute on Aging-Alzheimer’s Association workgroups on diagnostic guidelines for Alzheimer’s disease. Alzheimers Dement..

[50] Villemagne V.L., Okamura N. (2014). In vivo tau imaging: obstacles and progress. Alzheimers Dement..

[51] Okamura N., Harada R., Ishiki A., Kikuchi A., Nakamura T., Kudo Y. (2018). The development and validation of tau PET tracers: current status and future directions. Clin. Transl. Imaging.

[52] Aguero C., Dhaynaut M., Normandin M.D., Amaral A.C., Guehl N.J., Neelamegam R., Marquie M., Johnson K.A., El Fakhri G., Frosch M.P. (2019). Autoradiography validation of novel tau PET tracer F-18-MK-6240 on human postmortem brain tissue. Acta Neuropathol. Commun..

[53] Kroth H., Oden F., Molette J., Schieferstein H., Capotosti F., Mueller A., Berndt M., Schmitt-Willich H., Darmency V., Gabellieri E. (2019). Discovery and preclinical characterization of 18FPI-2620, a next-generation tau PET tracer for the assessment of tau pathology in Alzheimer’s disease and other tauopathies. Eur. J. Nucl. Med. Mol. Imaging.

[54] Spillantini M.G., Goedert M. (2000). The alpha-synucleinopathies: Parkinson’s disease, dementia with Lewy bodies, and multiple system atrophy. Ann. N. Y. Acad. Sci..

[55] Wakabayashi K., Yoshimoto M., Tsuji S., Takahashi H. (1998). α-Synuclein immunoreactivity in glial cytoplasmic inclusions in multiple system atrophy. Neuroscience Letters.

[56] Tu P.H., Galvin J.E., Baba M., Giasson B., Tomita T., Leight S., Nakajo S., Iwatsubo T., Trojanowski J.Q., Lee V.M. (1998). Glial cytoplasmic inclusions in white matter oligodendrocytes of multiple system atrophy brains contain insoluble alpha-synuclein. Ann. Neurol..

[57] Burré J., Sharma M., Tsetsenis T., Buchman V., Etherton M.R., Südhof T.C. (2010). Alpha-synuclein promotes SNARE-complex assembly in vivo and in vitro. Science.

[58] Vilar M., Chou H.-T., Lührs T., Maji S.K., Riek-Loher D., Verel R., Manning G., Stahlberg H., Riek R. (2008). The fold of alpha-synuclein fibrils. Proc. Natl. Acad. Sci. USA.

[59] Spillantini M.G., Crowther R.A., Jakes R., Hasegawa M., Goedert M. (1998). alpha-Synuclein in filamentous inclusions of Lewy bodies from Parkinson’s disease and dementia with lewy bodies. Proc. Natl. Acad. Sci. USA.

[60] Uéda K., Fukushima H., Masliah E., Xia Y., Iwai A., Yoshimoto M., Otero D.A., Kondo J., Ihara Y., Saitoh T. (1993). Molecular cloning of cDNA encoding an unrecognized component of amyloid in Alzheimer disease. Proc. Natl. Acad. Sci. USA.

[61] Clayton D.F., George J.M. (1999). Synucleins in synaptic plasticity and neurodegenerative disorders. J. Neurosci. Res..

[62] Polymeropoulos M.H., Lavedan C., Leroy E., Ide S.E., Dehejia A., Dutra A., Pike B., Root H., Rubenstein J., Boyer R. (1997). Mutation in the alpha-synuclein gene identified in families with Parkinson’s disease. Science.

[63] Chartier-Harlin M.-C., Kachergus J., Roumier C., Mouroux V., Douay X., Lincoln S., Levecque C., Larvor L., Andrieux J., Hulihan M. (2004). α-synuclein locus duplication as a cause of familial Parkinson’s disease. Lancet.

[64] Singleton A.B., Farrer M., Johnson J., Singleton A., Hague S., Kachergus J., Hulihan M., Peuralinna T., Dutra A., Nussbaum R. (2003). alpha-Synuclein locus triplication causes Parkinson’s disease. Science.

[65] Ibáñez P., Bonnet A.-M., Débarges B., Lohmann E., Tison F., Agid Y., Dürr A., Brice A., Pollak P. (2004). Causal relation between α-synuclein locus duplication as a cause of familial Parkinson’s disease. Lancet.

[66] Billingsley K.J., Bandres-Ciga S., Saez-Atienzar S., Singleton A.B. (2018). Genetic risk factors in Parkinson’s disease. Cell Tissue Res..

[67] Chang D., Nalls M.A., Hallgrímsdóttir I.B., Hunkapiller J., van der Brug M., Cai F., Kerchner G.A., Ayalon G., Bingol B., Sheng M. (2017). A meta-analysis of genome-wide association studies identifies 17 new Parkinson’s disease risk loci. Nat. Genet..

[68] Nalls M., Blauwendraat C., Vallerga C.L., Heilbron K., Bandres-Ciga S., Chang D., Tan M., Kia D.A., Noyce A.J., Xue A. Parkinson’s disease genetics: Novel risk loci, genomic context, causal insights and heritable risk. BiorRxiv.

[69] Vernon A.C., Ballard C., Modo M. (2010). Neuroimaging for Lewy body disease: is the in vivo molecular imaging of α-synuclein neuropathology required and feasible?. Brain Res. Rev..

[70] Hawkes C.H. (2008). The prodromal phase of sporadic Parkinson’s disease: does it exist and if so how long is it?. Mov. Disord..

[71] Ross G.W., Petrovitch H., Abbott R.D., Nelson J., Markesbery W., Davis D., Hardman J., Launer L., Masaki K., Tanner C.M. (2004). Parkinsonian signs and substantia nigra neuron density in decendents elders without PD. Ann. Neurol..

[72] Fearnley J.M., Lees A.J. (1991). Ageing and Parkinson’s disease: substantia nigra regional selectivity. Brain.

[73] Riederer P., Wuketich S. (1976). Time course of nigrostriatal degeneration in parkinson’s disease. A detailed study of influential factors in human brain amine analysis. J. Neural Transm..

[74] Braak H., Tredici K.D., Rüb U., de Vos R.A.I., Jansen Steur E.N.H., Braak E. (2003). Staging of brain pathology related to sporadic Parkinson’s disease. Neurobiol. Aging.

[75] Stefanis L. (2012). α-Synuclein in Parkinson’s disease. Cold Spring Harb. Perspect. Med..

[76] Deramecourt V., Bombois S., Maurage C.-A., Ghestem A., Drobecq H., Vanmechelen E., Lebert F., Pasquier F., Delacourte A. (2006). Biochemical staging of synucleinopathy and amyloid deposition in dementia with Lewy bodies. J. Neuropathol. Exp. Neurol..

[77] Shah M., Seibyl J., Cartier A., Bhatt R., Catafau A.M. (2014). Molecular imaging insights into neurodegeneration: focus on α-synuclein radiotracers. J. Nucl. Med..

[78] Barrett P.J., Timothy Greenamyre J. (2015). Post-translational modification of α-synuclein in Parkinson’s disease. Brain Res..

[79] Irwin D.J., Lee V.M.-Y., Trojanowski J.Q. (2013). Parkinson’s disease dementia: convergence of α-synuclein, tau and amyloid-β pathologies. Nat. Rev. Neurosci..

[80] Kotzbauer P.T., Cairns N.J., Campbell M.C., Willis A.W., Racette B.A., Tabbal S.D., Perlmutter J.S. (2012). Pathologic accumulation of α-synuclein and Aβ in Parkinson disease patients with dementia. Arch. Neurol..

[81] Mathis C.A., Lopresti B.J., Ikonomovic M.D., Klunk W.E. (2017). Small-molecule PET Tracers for Imaging Proteinopathies. Semin. Nucl. Med..

[82] Kotzbauer P.T., Tu Z., Mach R.H. (2017). Current status of the development of PET radiotracers for imaging alpha synuclein aggregates in Lewy bodies and Lewy neurites. Clin. Transl. Imaging.

[83] Pike V.W. (2016). Considerations in the Development of Reversibly Binding PET Radioligands for Brain Imaging. Curr. Med. Chem..

[84] Laruelle M., Slifstein M., Huang Y. (2003). Relationships between radiotracer properties and image quality in molecular imaging of the brain with positron emission tomography. Mol. Imaging Biol..

[85] Zhang L., Villalobos A. (2017). Strategies to facilitate the discovery of novel CNS PET ligands. EJNMMI Radiopharm. Chem..

[86] van de Bittner G.C., Ricq E.L., Hooker J.M. (2014). A philosophy for CNS radiotracer design. Acc. Chem. Res..

[87] Elghetany M.T., Saleem A. (1988). Methods for staining amyloid in tissues: A review. Stain Technol..

[88] Vassar P.S., Culling C.F. (1959). Fluorescent stains, with special reference to amyloid and connective tissues. Arch. Pathol..

[89] Watanabe H., Ono M., Ariyoshi T., Katayanagi R., Saji H. (2017). Novel Benzothiazole Derivatives as Fluorescent Probes for Detection of β-Amyloid and α-Synuclein Aggregates. ACS Chem. Neurosci..

[90] Zeng F., Goodman M.M. (2013). Fluorine-18 radiolabeled heterocycles as PET tracers for imaging β-amyloid plaques in Alzheimer’s disease. Curr. Top. Med. Chem..

[91] Mathis C.A., Mason N.S., Lopresti B.J., Klunk W.E. (2012). Development of positron emission tomography β-amyloid plaque imaging agents. Semin. Nucl. Med..

[92] Klunk W.E., Wang Y., Huang G.f., Debnath M.L., Holt D.P., Mathis C.A. (2001). Uncharged thioflavin-T derivatives bind to amyloid-beta protein with high affinity and readily enter the brain. Life Sciences.

[93] Klunk W.E., Wang Y., Huang G.-f., Debnath M.L., Holt D.P., Shao L., Hamilton R.L., Ikonomovic M.D., DeKosky S.T., Mathis C.A. (2003). The Binding of 2-(4′-Methylaminophenyl)Benzothiazole to Postmortem Brain Homogenates Is Dominated by the Amyloid Component. J. Neurosci..

[94] Mathis C.A., Wang Y., Holt D.P., Huang G.F., Debnath M.L., Klunk W.E. (2003). Synthesis and evaluation of 11C-labeled 6-substituted 2-arylbenzothiazoles as amyloid imaging agents. J. Med. Chem..

[95] Price J.C., Klunk W.E., Lopresti B.J., Lu X., Hoge J.A., Ziolko S.K., Holt D.P., Meltzer C.C., DeKosky S.T., Mathis C.A. (2005). Kinetic modeling of amyloid binding in humans using PET imaging and Pittsburgh Compound-B. J. Cereb. Blood Flow Metab..

[96] Mathis C.A., Bacskai B.J., Kajdasz S.T., McLellan M.E., Frosch M.P., Hyman B.T., Holt D.P., Wang Y., Huang G.-f., Debnath M.L. (2002). A lipophilic thioflavin-T derivative for positron emission tomography (PET) imaging of amyloid in brain. Bioorganic & Medicinal Chemistry Letters.

[97] Klunk W.E., Mathis C.A. (2008). Whatever happened to Pittsburgh Compound-A?. Alzheimer Dis. Assoc. Disord..

[98] Mintun M.A., Larossa G.N., Sheline Y.I., Dence C.S., Lee S.Y., Mach R.H., Klunk W.E., Mathis C.A., DeKosky S.T., Morris J.C. (2006). 11CPIB in a nondemented population: potential antecedent marker of Alzheimer disease. Neurology.

[99] Ikonomovic M.D., Klunk W.E., Abrahamson E.E., Mathis C.A., Price J.C., Tsopelas N.D., Lopresti B.J., Ziolko S., Bi W., Paljug W.R. (2008). Post-mortem correlates of in vivo PiB-PET amyloid imaging in a typical case of Alzheimer’s disease. Brain.

[100] Chitneni S.K., Serdons K., Evens N., Fonge H., Celen S., Deroose C.M., Debyser Z., Mortelmans L., Verbruggen A.M., Bormans G.M. (2008). Efficient purification and metabolite analysis of radiotracers using high-performance liquid chromatography and on-line solid-phase extraction. J. Chromatogr. A.

[101] Serdons K., Terwinghe C., Vermaelen P., van Laere K., Kung H., Mortelmans L., Bormans G., Verbruggen A. (2009). Synthesis and evaluation of 18F-labeled 2-phenylbenzothiazoles as positron emission tomography imaging agents for amyloid plaques in Alzheimer’s disease. J. Med. Chem..

[102] Mathis C.A., Holt D., Wang Y., Huang G.-f., Debnath M., Shao L., Klunk W.E. (2004). P2-178 Species-dependent formation and identification of the brain metabolites of the amyloid imaging agent [11C]PIB. Neurobiol. Aging.

[103] Juréus A., Swahn B.-M., Sandell J., Jeppsson F., Johnson A.E., Johnström P., Neelissen J.A.M., Sunnemark D., Farde L., Svensson S.P.S. (2010). Characterization of AZD4694, a novel fluorinated Abeta plaque neuroimaging PET radioligand. J. Neurochem..

[104] Mason N.S., Mathis C.A., Klunk W.E. (2013). Positron emission tomography radioligands for in vivo imaging of Aβ plaques. J. Labelled Comp. Radiopharm..

[105] Snellman A., Rokka J., Lopez-Picon F.R., Eskola O., Wilson I., Farrar G., Scheinin M., Solin O., Rinne J.O., Haaparanta-Solin M. (2012). Pharmacokinetics of ¹⁸Fflutemetamol in wild-type rodents and its binding to beta amyloid deposits in a mouse model of Alzheimer’s disease. Eur. J. Nucl. Med. Mol. Imaging.

[106] Rabinovici G.D. (2015). The translational journey of brain β-amyloid imaging: from positron emission tomography to autopsy to clinic. JAMA Neurol..

[107] Curtis C., Gamez J.E., Singh U., Sadowsky C.H., Villena T., Sabbagh M.N., Beach T.G., Duara R., Fleisher A.S., Frey K.A. (2015). Phase 3 trial of flutemetamol labeled with radioactive fluorine 18 imaging and neuritic plaque density. JAMA Neurol..

[108] GE Healthcare Gehealthcare Vizamyl Prescribing Information. http://www3.gehealthcare.com/~/media/documents/us-global/products/nuclear-imaging-agents_non-gatekeeper/clinical%20product%20info/vizamyl/gehealthcare-vizamyl-prescribing-information.pdf.

[109] Kung M.-P., Hou C., Zhuang Z.-P., Skovronsky D., Kung H.F. (2004). Binding of two potential imaging agents targeting amyloid plaques in postmortem brain tissues of patients with Alzheimer’s disease. Brain Res..

[110] Ono M., Wilson A., Nobrega J., Westaway D., Verhoeff P., Zhuang Z.-P., Kung M.-P., Kung H.F. (2003). 11C-labeled stilbene derivatives as Aβ-aggregate-specific PET imaging agents for Alzheimer’s disease. Nucl. Med. Biol..

[111] Verhoeff N.P.L.G., Wilson A.A., Takeshita S., Trop L., Hussey D., Singh K., Kung H.F., Kung M.-P., Houle S. (2004). In-vivo imaging of Alzheimer disease beta-amyloid with 11CSB-13 PET. Am. J. Geriatr. Psychiatry.

[112] Zhang W., Oya S., Kung M.-P., Hou C., Maier D.L., Kung H.F. (2005). F-18 stilbenes as PET imaging agents for detecting beta-amyloid plaques in the brain. J. Med. Chem..

[113] Stephenson K.A., Chandra R., Zhuang Z.-P., Hou C., Oya S., Kung M.-P., Kung H.F. (2007). Fluoro-pegylated (FPEG) imaging agents targeting Abeta aggregates. Bioconjug. Chem..

[114] Zhang W., Oya S., Kung M.-P., Hou C., Maier D.L., Kung H.F. (2005). F-18 Polyethyleneglycol stilbenes as PET imaging agents targeting Abeta aggregates in the brain. Nucl. Med. Biol..

[115] Kung H.F., Choi S.R., Qu W., Zhang W., Skovronsky D. (2010). 18F stilbenes and styrylpyridines for PET imaging of A beta plaques in Alzheimer’s disease: A miniperspective. J. Med. Chem..

[116] Rowe C.C., Ackerman U., Browne W., Mulligan R., Pike K.L., O’Keefe G., Tochon-Danguy H., Chan G., Berlangieri S.U., Jones G. (2008). Imaging of amyloid β in Alzheimer’s disease with 18F-BAY94-9172, a novel PET tracer: proof of mechanism. Lancet Neurol..

[117] Frequently Asked Questions about Beta-Amyloid Imaging. https://www.accessdata.fda.gov/drugsatfda_docs/label/2014/204677s000lbl.pdf.

[118] Zhang W., Kung M.-P., Oya S., Hou C., Kung H.F. (2007). 18F-labeled styrylpyridines as PET agents for amyloid plaque imaging. Nucl. Med. Biol..

[119] Choi S.R., Golding G., Zhuang Z., Zhang W., Lim N., Hefti F., Benedum T.E., Kilbourn M.R., Skovronsky D., Kung H.F. (2009). Preclinical properties of 18F-AV-45: A PET agent for Abeta plaques in the brain. J. Nucl. Med..

[120] Wong D.F., Rosenberg P.B., Zhou Y., Kumar A., Raymont V., Ravert H.T., Dannals R.F., Nandi A., Brasić J.R., Ye W. (2010). In vivo imaging of amyloid deposition in Alzheimer disease using the radioligand 18F-AV-45 (florbetapir corrected F 18). J. Nucl. Med..

[121] Okamura N., Yanai K. (2010). Florbetapir (18F), a PET imaging agent that binds to amyloid plaques for the potential detection of Alzheimer’s disease. IDrugs.

[122] Kolata G. Promise Seen for Detection of Alzheimer’s. https://www.nytimes.com/2010/06/24/health/research/24scans.html.

[123] Johnson A.E., Jeppsson F., Sandell J., Wensbo D., Neelissen J.A.M., Juréus A., Ström P., Norman H., Farde L., Svensson S.P.S. (2009). AZD2184: A radioligand for sensitive detection of beta-amyloid deposits. J. Neurochem..

[124] Swahn B.-M., Sandell J., Pyring D., Bergh M., Jeppsson F., Juréus A., Neelissen J., Johnström P., Schou M., Svensson S. (2012). Synthesis and evaluation of pyridylbenzofuran, pyridylbenzothiazole and pyridylbenzoxazole derivatives as ¹⁸F-PET imaging agents for β-amyloid plaques. Bioorg. Med. Chem. Lett..

[125] Nelissen N., van Laere K., Thurfjell L., Owenius R., Vandenbulcke M., Koole M., Bormans G., Brooks D.J., Vandenberghe R. (2009). Phase 1 study of the Pittsburgh compound B derivative 18F-flutemetamol in healthy volunteers and patients with probable Alzheimer disease. J. Nucl. Med..

[126] Mathis C., Lopresti B., Mason N., Price J., Flatt N., Bi W., Ziolko S., DeKosky S., Klunk W. Comparison of the Amyloid Imaging Agents [F-18]3′-F-PIB and [C-11]PIB in Alzheimer’s Disease and Control Subjects. http://jnm.snmjournals.org/content/48/supplement_2/56P.3.short.

[127] Lopresti B.J., Klunk W.E., Mathis C.A., Hoge J.A., Ziolko S.K., Lu X., Meltzer C.C., Schimmel K., Tsopelas N.D., DeKosky S.T. (2005). Simplified quantification of Pittsburgh Compound B amyloid imaging PET studies: A comparative analysis. J. Nucl. Med..

[128] Cselényi Z., Jönhagen M.E., Forsberg A., Halldin C., Julin P., Schou M., Johnström P., Varnäs K., Svensson S., Farde L. (2012). Clinical validation of 18F-AZD4694, an amyloid-β-specific PET radioligand. J. Nucl. Med..

[129] A Phase 3 Clinical Trial to Evaluate the Efficacy and Safety of [18F]NAV4694 PET for Detection of Cerebral Beta-Amyloid When Compared With Postmortem Histopathology-Full Text View-ClinicalTrials.gov. https://clinicaltrials.gov/ct2/show/NCT01886820.

[130] Flutafuranol F 18-Navidea Biopharmaceuticals-AdisInsight. https://adisinsight.springer.com/drugs/800031066.

[131] Hostetler E.D., Sanabria-Bohórquez S., Fan H., Zeng Z., Gammage L., Miller P., O’Malley S., Connolly B., Mulhearn J., Harrison S.T. (2011). 18FFluoroazabenzoxazoles as potential amyloid plaque PET tracers: synthesis and in vivo evaluation in rhesus monkey. Nucl. Med. Biol..

[132] Merck Clinical Trials. https://www.merck.com/clinical-trials/study.html?id=3328-002&kw=alzheimer%27s&tab=eligibility.

[133] Yousefi B.H., Drzezga A., von Reutern B., Manook A., Schwaiger M., Wester H.-J., Henriksen G. (2011). A Novel (18)F-Labeled Imidazo2,1-bbenzothiazole (IBT) for High-Contrast PET Imaging of β-Amyloid Plaques. ACS Med. Chem. Lett..

[134] Yousefi B.H., von Reutern B., Scherübl D., Manook A., Schwaiger M., Grimmer T., Henriksen G., Förster S., Drzezga A., Wester H.-J. (2015). FIBT versus florbetaben and PiB: A preclinical comparison study with amyloid-PET in transgenic mice. EJNMMI Res..

[135] Westwell A.D. (2015). Fluorinated Pharmaceuticals. Development of 18F-labeled compounds for imaging of Aβ plaques by means of PET..

[136] Yousefi B.H., Manook A., Grimmer T., Arzberger T., von Reutern B., Henriksen G., Drzezga A., Förster S., Schwaiger M., Wester H.-J. (2015). Characterization and first human investigation of FIBT, a novel fluorinated Aβ plaque neuroimaging PET radioligand. ACS Chem. Neurosci..

[137] Kim Y., Rosenberg P., Oh E. (2018). A Review of Diagnostic Impact of Amyloid Positron Emission Tomography Imaging in Clinical Practice. Dement. Geriatr. Cogn. Disord..

[138] Barthel H., Sabri O. (2017). Clinical Use and Utility of Amyloid Imaging. J. Nucl. Med..

[139] Chiotis K., Saint-Aubert L., Boccardi M., Gietl A., Picco A., Varrone A., Garibotto V., Herholz K., Nobili F., Nordberg A. (2017). Clinical validity of increased cortical uptake of amyloid ligands on PET as a biomarker for Alzheimer’s disease in the context of a structured 5-phase development framework. Neurobiol. Aging.

[140] Ceccaldi M., Jonveaux T., Verger A., Salmon P.K., Houzard C., Godefroy O., Shields T., Perrotin A., Gismondi R., Bullich S. Impact of Florbetaben PET Imaging on Diagnosis and Management of Patients with Suspected Alzheimer’s Disease Eligible for CSF Analysis in France. http://jnm.snmjournals.org/content/58/supplement_1/561?related-urls=yes&legid=jnumed;58/supplement_1/561.

[141] Hattori N., Ono S., UDO N., Yamamoto S., Ogawa M., Sugie H. Clinical Impact of F-18 Flutemetamol (FMM) PET to Assess Cerebral Aß Pathology in Patients with Various Cognitive Disorders. http://jnm.snmjournals.org/content/59/supplement_1/480.

[142] Leuzy A., Savitcheva I., Chiotis K., Lilja J., Andersen P., Bogdanovic N., Jelic V., Nordberg A. (2019). Clinical impact of 18Fflutemetamol PET among memory clinic patients with an unclear diagnosis. Eur. J. Nucl. Med. Mol. Imaging.

[143] Sabri O., Seibyl J., Rowe C., Barthel H. (2015). Beta-amyloid imaging with florbetaben. Clin. Transl. Imaging.

[144] de Wilde A., van der Flier W.M., Pelkmans W., Bouwman F., Verwer J., Groot C., van Buchem M.M., Zwan M., Ossenkoppele R., Yaqub M. (2018). Association of Amyloid Positron Emission Tomography With Changes in Diagnosis and Patient Treatment in an Unselected Memory Clinic Cohort: The ABIDE Project. JAMA Neurol..

[145] Zannas A.S., Doraiswamy P.M., Shpanskaya K.S., Murphy K.R., Petrella J.R., Burke J.R., Wong T.Z. (2014). Impact of ¹⁸F-florbetapir PET imaging of β-amyloid neuritic plaque density on clinical decision-making. Neurocase.

[146] University of Zurich. Investigating the Clinical Consequences of Flutemetamol-PET-Scanning-ICH GCP-Clinical Trials Registry. https://ichgcp.net/clinical-trials-registry/NCT02353949.

[147] Buée L., Bussière T., Buée-Scherrer V., Delacourte A., Hof P.R. (2000). Tau protein isoforms, phosphorylation and role in neurodegenerative disorders11These authors contributed equally to this work. Brain Res. Rev..

[148] Fichou Y., Al-Hilaly Y.K., Devred F., Smet-Nocca C., Tsvetkov P.O., Verelst J., Winderickx J., Geukens N., Vanmechelen E., Perrotin A. (2019). The elusive tau molecular structures: can we translate the recent breakthroughs into new targets for intervention?. Acta Neuropathol. Commun..

[149] Harada R., Okamura N., Furumoto S., Tago T., Yanai K., Arai H., Kudo Y. (2016). Characteristics of Tau and Its Ligands in PET Imaging. Biomolecules.

[150] Mott R.T., Dickson D.W., Trojanowski J.Q., Zhukareva V., Lee V.M., Forman M., van Deerlin V., Ervin J.F., Wang D.-S., Schmechel D.E. (2005). Neuropathologic, biochemical, and molecular characterization of the frontotemporal dementias. J. Neuropathol. Exp. Neurol..

[151] Dickson D.W., Kouri N., Murray M.E., Josephs K.A. (2011). Neuropathology of frontotemporal lobar degeneration-tau (FTLD-tau). J. Mol. Neurosci..

[152] Harada R., Okamura N., Furumoto S., Yanai K. (2018). Imaging Protein Misfolding in the Brain Using β-Sheet Ligands. Front. Neurosci..

[153] Clinton L.K., Blurton-Jones M., Myczek K., Trojanowski J.Q., LaFerla F.M. (2010). Synergistic Interactions between Abeta, tau, and alpha-synuclein: Acceleration of neuropathology and cognitive decline. J. Neurosci..

[154] Näslund J., Haroutunian V., Mohs R., Davis K.L., Davies P., Greengard P., Buxbaum J.D. (2000). Correlation between elevated levels of amyloid beta-peptide in the brain and cognitive decline. JAMA.

[155] Okamura N., Suemoto T., Furumoto S., Suzuki M., Shimadzu H., Akatsu H., Yamamoto T., Fujiwara H., Nemoto M., Maruyama M. (2005). Quinoline and benzimidazole derivatives: candidate probes for in vivo imaging of tau pathology in Alzheimer’s disease. J. Neurosci..

[156] Fodero-Tavoletti M.T., Okamura N., Furumoto S., Mulligan R.S., Connor A.R., McLean C.A., Cao D., Rigopoulos A., Cartwright G.A., O’Keefe G. (2011). 18F-THK523: A novel in vivo tau imaging ligand for Alzheimer’s disease. Brain.

[157] Okamura N., Furumoto S., Harada R., Tago T., Yoshikawa T., Fodero-Tavoletti M., Mulligan R.S., Villemagne V.L., Akatsu H., Yamamoto T. (2013). Novel 18F-labeled arylquinoline derivatives for noninvasive imaging of tau pathology in Alzheimer disease. J. Nucl. Med..

[158] Villemagne V.L., Furumoto S., Fodero-Tavoletti M.T., Mulligan R.S., Hodges J., Harada R., Yates P., Piguet O., Pejoska S., Doré V. (2014). In vivo evaluation of a novel tau imaging tracer for Alzheimer’s disease. Eur. J. Nucl. Med. Mol. Imaging.

[159] Tago T., Furumoto S., Okamura N., Harada R., Adachi H., Ishikawa Y., Yanai K., Iwata R., Kudo Y. (2016). Preclinical Evaluation of (18)FTHK-5105 Enantiomers: Effects of Chirality on Its Effectiveness as a Tau Imaging Radiotracer. Mol. Imaging Biol..

[160] Okamura N., Furumoto S., Fodero-Tavoletti M.T., Mulligan R.S., Harada R., Yates P., Pejoska S., Kudo Y., Masters C.L., Yanai K. (2014). Non-invasive assessment of Alzheimer’s disease neurofibrillary pathology using 18F-THK5105 PET. Brain.

[161] Saint-Aubert L., Almkvist O., Chiotis K., Almeida R., Wall A., Nordberg A. (2016). Regional tau deposition measured by 18FTHK5317 positron emission tomography is associated to cognition via glucose metabolism in Alzheimer’s disease. Alzheimers Res. Ther..

[162] Chiotis K., Saint-Aubert L., Savitcheva I., Jelic V., Andersen P., Jonasson M., Eriksson J., Lubberink M., Almkvist O., Wall A. (2016). Imaging in-vivo tau pathology in Alzheimer’s disease with THK5317 PET in a multimodal paradigm. Eur. J. Nucl. Med. Mol. Imaging.

[163] Tago T., Furumoto S., Okamura N., Harada R., Adachi H., Ishikawa Y., Yanai K., Iwata R., Kudo Y. (2016). Structure-Activity Relationship of 2-Arylquinolines as PET Imaging Tracers for Tau Pathology in Alzheimer Disease. J. Nucl. Med..

[164] Harada R., Okamura N., Furumoto S., Furukawa K., Ishiki A., Tomita N., Tago T., Hiraoka K., Watanuki S., Shidahara M. (2016). 18F-THK5351: A Novel PET Radiotracer for Imaging Neurofibrillary Pathology in Alzheimer Disease. J. Nucl. Med..

[165] Sone D., Imabayashi E., Maikusa N., Okamura N., Furumoto S., Kudo Y., Ogawa M., Takano H., Yokoi Y., Sakata M. (2017). Regional tau deposition and subregion atrophy of medial temporal structures in early Alzheimer’s disease: A combined positron emission tomography/magnetic resonance imaging study. Alzheimers Dement..

[166] Betthauser T.J., Lao P.J., Murali D., Barnhart T.E., Furumoto S., Okamura N., Stone C.K., Johnson S.C., Christian B.T. (2017). In Vivo Comparison of Tau Radioligands 18F-THK-5351 and 18F-THK-5317. J. Nucl. Med..

[167] Edmondson D.E., Mattevi A., Binda C., Li M., Hubálek F. (2004). Structure and mechanism of monoamine oxidase. Curr. Med. Chem..

[168] Tipton K.F., Boyce S., O’Sullivan J., Davey G.P., Healy J. (2004). Monoamine oxidases: certainties and uncertainties. Curr. Med. Chem..

[169] Ishiki A., Harada R., Kai H., Sato N., Totsune T., Tomita N., Watanuki S., Hiraoka K., Ishikawa Y., Funaki Y. (2018). Neuroimaging-pathological correlations of 18FTHK5351 PET in progressive supranuclear palsy. Acta Neuropathol. Commun..

[170] Harada R., Ishiki A., Kai H., Sato N., Furukawa K., Furumoto S., Tago T., Tomita N., Watanuki S., Hiraoka K. (2018). Correlations of 18F-THK5351 PET with Postmortem Burden of Tau and Astrogliosis in Alzheimer Disease. J. Nucl. Med..

[171] Krebs M.R.H., Bromley E.H.C., Donald A.M. (2005). The binding of thioflavin-T to amyloid fibrils: localisation and implications. J. Struct. Biol..

[172] Maruyama M., Shimada H., Suhara T., Shinotoh H., Ji B., Maeda J., Zhang M.-R., Trojanowski J.Q., Lee V.M.-Y., Ono M. (2013). Imaging of tau pathology in a tauopathy mouse model and in Alzheimer patients compared to normal controls. Neuron.

[173] Hashimoto H., Kawamura K., Igarashi N., Takei M., Fujishiro T., Aihara Y., Shiomi S., Muto M., Ito T., Furutsuka K. (2014). Radiosynthesis, photoisomerization, biodistribution, and metabolite analysis of 11C-PBB3 as a clinically useful PET probe for imaging of tau pathology. J. Nucl. Med..

[174] Koga S., Ono M., Sahara N., Higuchi M., Dickson D.W. (2017). Fluorescence and autoradiographic evaluation of tau PET ligand PBB3 to α-synuclein pathology. Mov. Disord..

[175] Perez-Soriano A., Arena J.E., Dinelle K., Miao Q., McKenzie J., Neilson N., Puschmann A., Schaffer P., Shinotoh H., Smith-Forrester J. (2017). PBB3 imaging in Parkinsonian disorders: Evidence for binding to tau and other proteins. Mov. Disord..

[176] Hsieh C.-J., Mach R.H., Zhude T., Kotzbauer P.T. (2018). Imaging of Aggregated Alpha-Synuclein in Parkinson’s Disease: A Work in Progress. The newsletter of the SNMI. Centrer for Molecular Imaging Innovation and Translation.

[177] Cashion D.K., Chen G., Kasi D., Kolb H.C., Liu C., Sinha A., Szardenings A.K., Wang E., Yu C., Zhang W. Imaging Agents for Detecting Neurological Disorders. https://patentscope.wipo.int/search/en/detail.jsf?docId=WO2011119565.

[178] Xia C.-F., Arteaga J., Chen G., Gangadharmath U., Gomez L.F., Kasi D., Lam C., Liang Q., Liu C., Mocharla V.P. (2013). (18)FT807, a novel tau positron emission tomography imaging agent for Alzheimer’s disease. Alzheimers Dement..

[179] Zhang W., Arteaga J., Cashion D.K., Chen G., Gangadharmath U., Gomez L.F., Kasi D., Lam C., Liang Q., Liu C. (2012). A highly selective and specific PET tracer for imaging of tau pathologies. J. Alzheimers Dis..

[180] Chien D.T., Bahri S., Szardenings A.K., Walsh J.C., Mu F., Su M.-Y., Shankle W.R., Elizarov A., Kolb H.C. (2013). Early clinical PET imaging results with the novel PHF-tau radioligand F-18-T807. J. Alzheimers Dis..

[181] Mintun M., Schwarz A., Joshi A., Shcherbinin S., Chien D., Elizarov A., Su M.-Y., Shankle W., Pontecorvo M., Tauscher J. (2013). Exploratory analyses of regional human brain distribution of the PET tau tracer F18-labeled T807 (AV-1541) in subjects with normal cognitive function or cognitive impairment thought to be due to Alzheimer’s disease. Alzheimer’s Dement..

[182] Ono M., Sahara N., Kumata K., Ji B., Ni R., Koga S., Dickson D.W., Trojanowski J.Q., Lee V.M.-Y., Yoshida M. (2017). Distinct binding of PET ligands PBB3 and AV-1451 to tau fibril strains in neurodegenerative tauopathies. Brain.

[183] Cho H., Choi J.Y., Lee S.H., Ryu Y.H., Lee M.S., Lyoo C.H. (2017). 18 F-AV-1451 binds to putamen in multiple system atrophy. Mov. Disord..

[184] Leuzy A., Chiotis K., Lemoine L., Gillberg P.-G., Almkvist O., Rodriguez-Vieitez E., Nordberg A. (2019). Tau PET imaging in neurodegenerative tauopathies-still a challenge. Mol. Psychiatry.

[185] Kolb H.C., Andrés J.I. (2017). Tau Positron Emission Tomography Imaging. Cold Spring Harb. Perspect. Biol..

[186] Lowe V.J., Curran G., Fang P., Liesinger A.M., Josephs K.A., Parisi J.E., Kantarci K., Boeve B.F., Pandey M.K., Bruinsma T. (2016). An autoradiographic evaluation of AV-1451 Tau PET in dementia. Acta Neuropathol. Commun..

[187] Hostetler E.D., Walji A.M., Zeng Z., Miller P., Bennacef I., Salinas C., Connolly B., Gantert L., Haley H., Holahan M. (2016). Preclinical Characterization of 18F-MK-6240, a Promising PET Tracer for In Vivo Quantification of Human Neurofibrillary Tangles. J. Nucl. Med..

[188] Vermeiren C., Motte P., Viot D., Mairet-Coello G., Courade J.-P., Citron M., Mercier J., Hannestad J., Gillard M. (2018). The tau positron-emission tomography tracer AV-1451 binds with similar affinities to tau fibrils and monoamine oxidases. Mov. Disord..

[189] Wooten D.W., Guehl N.J., Verwer E.E., Shoup T.M., Yokell D.L., Zubcevik N., Vasdev N., Zafonte R.D., Johnson K.A., El Fakhri G. (2017). Pharmacokinetic Evaluation of the Tau PET Radiotracer 18F-T807 (18F-AV-1451) in Human Subjects. J. Nucl. Med..

[190] Shcherbinin S., Schwarz A.J., Joshi A., Navitsky M., Flitter M., Shankle W.R., Devous M.D., Mintun M.A. (2016). Kinetics of the Tau PET Tracer 18F-AV-1451 (T807) in Subjects with Normal Cognitive Function, Mild Cognitive Impairment, and Alzheimer Disease. J. Nucl. Med..

[191] Declercq L., Celen S., Lecina J., Ahamed M., Tousseyn T., Moechars D., Alcazar J., Ariza M., Fierens K., Bottelbergs A. (2016). Comparison of New Tau PET-Tracer Candidates With 18FT808 and 18FT807. Mol. Imaging.

[192] Wang Y.T., Edison P. (2019). Tau Imaging in Neurodegenerative Diseases Using Positron Emission Tomography. Curr. Neurol. Neurosci. Rep..

[193] Sanabria Bohórquez S., Marik J., Ogasawara A., Tinianow J.N., Gill H.S., Barret O., Tamagnan G., Alagille D., Ayalon G., Manser P. (2019). 18FGTP1 (Genentech Tau Probe 1), a radioligand for detecting neurofibrillary tangle tau pathology in Alzheimer’s disease. Eur. J. Nucl. Med. Mol. Imaging.

[194] Barret O., Alagille D., Sanabria S., Comley R.A., Weimer R.M., Borroni E., Mintun M., Seneca N., Papin C., Morley T. (2017). Kinetic Modeling of the Tau PET Tracer 18F-AV-1451 in Human Healthy Volunteers and Alzheimer Disease Subjects. J. Nucl. Med..

[195] Seki C., Tagai K., Shimada H., Takahata K., Kubota M., Takado Y., Shinitoh H., Kimura Y., Ichise M., Okada M. (2019). Establishment of a Simplified Method to Quantify [18F]PM-PBB3 ([18F]APN-1607) Binding in the Brains of Living Human Subjects. https://repo.qst.go.jp/?action=pages_view_main&active_action=repository_view_main_item_detail&item_id=78239&item_no=1&page_id=13&block_id=21.

[196] 18F-PM-PBB3 PET Study in Tauopathy Including Alzheimer’s Disease, Other Dementias and Normal Controls-Full Text View-ClinicalTrials.gov. https://clinicaltrials.gov/ct2/show/study/NCT03625128.

[197] Brendel M., Yousefi B.H., Blume T., Herz M., Focke C., Deussing M., Peters F., Lindner S., von Ungern-Sternberg B., Drzezga A. (2018). Comparison of 18F-T807 and 18F-THK5117 PET in a Mouse Model of Tau Pathology. Front. Aging Neurosci..

[198] Wong D.F., Comley R.A., Kuwabara H., Rosenberg P.B., Resnick S.M., Ostrowitzki S., Vozzi C., Boess F., Oh E., Lyketsos C.G. (2018). Characterization of 3 Novel Tau Radiopharmaceuticals, 11C-RO-963, 11C-RO-643, and 18F-RO-948, in Healthy Controls and in Alzheimer Subjects. J. Nucl. Med..

[199] Gobbi L.C., Knust H., Körner M., Honer M., Czech C., Belli S., Muri D., Edelmann M.R., Hartung T., Erbsmehl I. (2017). Identification of Three Novel Radiotracers for Imaging Aggregated Tau in Alzheimer’s Disease with Positron Emission Tomography. J. Med. Chem..

[200] Honer M., Gobbi L., Knust H., Kuwabara H., Muri D., Koerner M., Valentine H., Dannals R.F., Wong D.F., Borroni E. (2018). Preclinical Evaluation of 18F-RO6958948, 11C-RO6931643, and 11C-RO6924963 as Novel PET Radiotracers for Imaging Tau Aggregates in Alzheimer Disease. J. Nucl. Med..

[201] Marquié M., Normandin M.D., Vanderburg C.R., Costantino I.M., Bien E.A., Rycyna L.G., Klunk W.E., Mathis C.A., Ikonomovic M.D., Debnath M.L. (2015). Validating novel tau positron emission tomography tracer F-18-AV-1451 (T807) on postmortem brain tissue. Ann. Neurol..

[202] Walji A.M., Hostetler E.D., Selnick H., Zeng Z., Miller P., Bennacef I., Salinas C., Connolly B., Gantert L., Holahan M. (2016). Discovery of 6-(Fluoro-(18)F)-3-(1H-pyrrolo2,3-cpyridin-1-yl)isoquinolin-5-amine ((18)F-MK-6240): A Positron Emission Tomography (PET) Imaging Agent for Quantification of Neurofibrillary Tangles (NFTs). J. Med. Chem..

[203] Pascoal T.A., Shin M., Kang M.S., Chamoun M., Chartrand D., Mathotaarachchi S., Bennacef I., Therriault J., Ng K.P., Hopewell R. (2018). In vivo quantification of neurofibrillary tangles with 18FMK-6240. Alzheimers Res. Ther..

[204] Betthauser T.J., Cody K.A., Zammit M.D., Murali D., Converse A.K., Barnhart T.E., Stone C.K., Rowley H.A., Johnson S.C., Christian B.T. (2019). In Vivo Characterization and Quantification of Neurofibrillary Tau PET Radioligand 18F-MK-6240 in Humans from Alzheimer Disease Dementia to Young Controls. J. Nucl. Med..

[205] [18F]MK-6240 Positron Emission Tomography (PET) Tracer First-in-Human Validation Study (MK-6240-001)-Full Text View-ClinicalTrials.gov. https://clinicaltrials.gov/ct2/show/NCT02562989.

[206] Declercq L., Rombouts F., Koole M., Fierens K., Mariën J., Langlois X., Andrés J.I., Schmidt M., Macdonald G., Moechars D. (2017). Preclinical Evaluation of 18F-JNJ64349311, a Novel PET Tracer for Tau Imaging. J. Nucl. Med..

[207] Politis M. (2014). Neuroimaging in Parkinson disease: from research setting to clinical practice. Nat. Rev. Neurol..

[208] Dickson D.W., Braak H., Duda J.E., Duyckaerts C., Gasser T., Halliday G.M., Hardy J., Leverenz J.B., Del Tredici K., Wszolek Z.K. (2009). Neuropathological assessment of Parkinson’s disease: refining the diagnostic criteria. Lancet Neurol..

[209] Yu L., Cui J., Padakanti P.K., Engel L., Bagchi D.P., Kotzbauer P.T., Tu Z. (2012). Synthesis and in vitro evaluation of α-synuclein ligands. Bioorg. Med. Chem..

[210] Bagchi D.P., Yu L., Perlmutter J.S., Xu J., Mach R.H., Tu Z., Kotzbauer P.T. (2013). Binding of the radioligand SIL23 to α-synuclein fibrils in Parkinson disease brain tissue establishes feasibility and screening approaches for developing a Parkinson disease imaging agent. PLoS ONE.

[211] Zhang X., Jin H., Padakanti P.K., Li J., Yang H., Fan J., Mach R.H., Kotzbauer P., Tu Z. (2014). Radiosynthesis and in Vivo Evaluation of Two PET Radioligands for Imaging α-Synuclein. Appl. Sci..

[212] Honson N.S., Johnson R.L., Huang W., Inglese J., Austin C.P., Kuret J. (2007). Differentiating Alzheimer disease-associated aggregates with small molecules. Neurobiol. Dis..

[213] Chu W., Zhou D., Gaba V., Liu J., Li S., Peng X., Xu J., Dhavale D., Bagchi D.P., d’Avignon A. (2015). Design, Synthesis, and Characterization of 3-(Benzylidene)indolin-2-one Derivatives as Ligands for α-Synuclein Fibrils. J. Med. Chem..

[214] Hsieh C.-J., Xu K., Lee I., Graham T.J.A., Tu Z., Dhavale D., Kotzbauer P., Mach R.H. (2018). Chalcones and Five-Membered Heterocyclic Isosteres Bind to Alpha Synuclein Fibrils in Vitro. ACS Omega.

[215] Ono M., Maya Y., Haratake M., Ito K., Mori H., Nakayama M. (2007). Aurones serve as probes of beta-amyloid plaques in Alzheimer’s disease. Biochem. Biophys. Res. Commun..

[216] Ono M., Haratake M., Mori H., Nakayama M. (2007). Novel chalcones as probes for in vivo imaging of beta-amyloid plaques in Alzheimer’s brains. Bioorg. Med. Chem..

[217] Ono M., Yoshida N., Ishibashi K., Haratake M., Arano Y., Mori H., Nakayama M. (2005). Radioiodinated flavones for in vivo imaging of beta-amyloid plaques in the brain. J. Med. Chem..

[218] Meng X., Munishkina L.A., Fink A.L., Uversky V.N. (2010). Effects of Various Flavonoids on the α-Synuclein Fibrillation Process. Parkinsons. Dis..

[219] Zhu M., Han S., Fink A.L. (2013). Oxidized quercetin inhibits α-synuclein fibrillization. Biochim. Biophys. Acta.

[220] Masuda M., Suzuki N., Taniguchi S., Oikawa T., Nonaka T., Iwatsubo T., Hisanaga S.-i., Goedert M., Hasegawa M. (2006). Small molecule inhibitors of alpha-synuclein filament assembly. Biochemistry.

[221] Cui M., Ono M., Watanabe H., Kimura H., Liu B., Saji H. (2014). Smart near-infrared fluorescence probes with donor-acceptor structure for in vivo detection of β-amyloid deposits. J. Am. Chem. Soc..

[222] Ono M., Doi Y., Watanabe H., Ihara M., Ozaki A., Saji H. (2016). Structure–activity relationships of radioiodinated diphenyl derivatives with different conjugated double bonds as ligands for α-synuclein aggregates. RSC Adv..

[223] Fanti S., Bonfiglioli R., Decristoforo C. (2018). Highlights of the 30th Annual Congress of the EANM, Vienna 2017: “Yes we can-make nuclear medicine great again”. Eur. J. Nucl. Med. Mol. Imaging.

[224] Wester H.-J., Yousefi B.H. US20170157274A1-Compounds Binding to Neuropathological Aggregates-Google Patents. https://patents.google.com/patent/US20170157274A1/en.

